# Cannabinoid Modulation of Dopamine Release During Motivation, Periodic Reinforcement, Exploratory Behavior, Habit Formation, and Attention

**DOI:** 10.3389/fnsyn.2021.660218

**Published:** 2021-06-10

**Authors:** Erik B. Oleson, Lindsey R. Hamilton, Devan M. Gomez

**Affiliations:** ^1^Department of Psychology, University of Colorado Denver, Denver, CO, United States; ^2^Department of Biomedical Sciences, Marquette University, Milwaukee, WI, United States

**Keywords:** cannabinoids, dopamine, motivation, reinforcement, attention, habit, timing, adjunctive

## Abstract

Motivational and attentional processes energize action sequences to facilitate evolutionary competition and promote behavioral fitness. Decades of neuropharmacology, electrophysiology and electrochemistry research indicate that the mesocorticolimbic DA pathway modulates both motivation and attention. More recently, it was realized that mesocorticolimbic DA function is tightly regulated by the brain’s endocannabinoid system and greatly influenced by exogenous cannabinoids—which have been harnessed by humanity for medicinal, ritualistic, and recreational uses for 12,000 years. Exogenous cannabinoids, like the primary psychoactive component of cannabis, delta-9-tetrahydrocannabinol, produce their effects by acting at binding sites for naturally occurring endocannabinoids. The brain’s endocannabinoid system consists of two G-protein coupled receptors, endogenous lipid ligands for these receptor targets, and several synthetic and metabolic enzymes involved in their production and degradation. Emerging evidence indicates that the endocannabinoid 2-arachidonoylglycerol is necessary to observe concurrent increases in DA release and motivated behavior. And the historical pharmacology literature indicates a role for cannabinoid signaling in both motivational and attentional processes. While both types of behaviors have been scrutinized under manipulation by either DA or cannabinoid agents, there is considerably less insight into prospective interactions between these two important signaling systems. This review attempts to summate the relevance of cannabinoid modulation of DA release during operant tasks designed to investigate either motivational or attentional control of behavior. We first describe how cannabinoids influence DA release and goal-directed action under a variety of reinforcement contingencies. Then we consider the role that endocannabinoids might play in switching an animal’s motivation from a goal-directed action to the search for an alternative outcome, in addition to the formation of long-term habits. Finally, dissociable features of attentional behavior using both the 5-choice serial reaction time task and the attentional set-shifting task are discussed along with their distinct influences by DA and cannabinoids. We end with discussing potential targets for further research regarding DA-cannabinoid interactions within key substrates involved in motivation and attention.

## Introduction

### Statement of Purpose

Because many reviews already exist that describe endocannabinoid (eCB) signaling (Toczek and Malinowska, 2018; Zou and Kumar, 2018; Cristino et al., 2020; Kaczocha and Haj-Dahmane, 2021), the risks of cannabis abuse (Ferland and Hurd, 2020; Hindley et al., 2020), and the potential cannabinoids may offer in psychiatric medicine (Amar, 2006; Black et al., 2019; Navarrete et al., 2020), our goal in the present manuscript is to describe how exogenous cannabinoids and eCBs influence dopamine (DA) signaling and behavior. While we will emphasize our own observations, we will also consider how they fit into the context of the general literature on reinforcement, appetitive behavior, adjunctive behavior, habit formation, and attentional processes. We conclude by considering how cannabinoid-induced changes in one neurobehavior might influence another if they share overlapping neural circuitry.

### Phytocannabinoids, Synthetic Cannabinoids, Endocannabinoids

Approximately 535 chemicals and 90 different C_21_ terpenophenolic phytocannabinoids exist in the cannabis plant (Radwan et al., 2009; Andre et al., 2016). While these chemicals act synergistically to produce an entourage effect with delta-9-tetrahydrocannabinol (THC), the latter is principally responsible for cannabis’s psychoactive effects by activating G protein-coupled receptors (GPCR) in the brain (e.g., cannabinoid receptor type 1; CB1) (Casajuana Kögel et al., 2018; Russo, 2019). The first synthetic cannabinoids (e.g., CP-55,940) were developed by Pfizer and found to be more potent and effective at activating the CB1 than THC (Matsuda et al., 1990; Marzo and Petrocellis, 2006). The Sterling research group then discovered that uniquely structured aminoalkylindole agonists also activate the CB1 with high potency and efficacy (D’Ambra et al., 1992). The aminoalkylindole synthetic cannabinoid WIN 55,212-2 (WIN) is particularly noteworthy because it has been employed extensively in psychopharmacology research (D’Ambra et al., 1992)—including several studies that will be described herein. However, it should be noted that WIN is about 80% more effective at activating the CB1 than the phytocannabinoid THC (Sim et al., 1996). The discovery of a brain cannabinoid receptor led to an exploration for its endogenous ligands, or eCBs (Marzo and Petrocellis, 2006). The best characterized eCBs are 2-arachidonoylglycerol (2AG) (Mechoulam et al., 1995; Sugiura et al., 1995) and *N*-arachidonoylethanolamine (anandamide; AEA) (Devane et al., 1992). It is now recognized that 2AG and AEA have different synthetic and metabolic pathways (Lu and Mackie, 2016). 2AG is predominantly synthesized from 2-arachidonoyl-containing phospholipids (e.g., diacylglycerol; DAG) by DAG lipase (DAGL) and metabolized by monoacylglycerol lipase (MAGL); AEA is predominantly synthesized from *N*-acyl-phosphatidylethanolamine (NAPE) by NAPE-specific phospholipase D (NAPE-PLD) and metabolized by fatty acid amidohydrolase (FAAH) (Lu and Mackie, 2016; Toczek and Malinowska, 2018; Zou and Kumar, 2018). In this review, we will describe several studies that manipulate 2AG levels by inhibiting either DAGL or MAGL. We attempt to specify when we are describing the specific effects of phytocannabinoids, synthetic cannabinoids, or eCBs on neurobiology and behavior. When making broader conclusions we use the term cannabinoid, which we define as any ligand that interacts with the cannabinoid receptors or their associated machinery.

### Cannabinoid Receptors

In addition to the aforementioned CB1 it is necessary to acknowledge several other cannabinoid receptor targets, most notably the cannabinoid receptor type 2 (CB2). While once thought to be relegated to the immune system and spleen, recent evidence suggest that CB2 is expressed in both neurons and glial cells of the brain as a unique isoform (Jordan and Xi, 2019). Specifically, mRNA for the CB_2A_ variant was found to be expressed in the brain and the testis, whereas mRNA for the CB_2B_ variant was found in the spleen and immune cells (Liu et al., 2009). The exact role that the CB_2A_ variant plays in modulating operant behavior remains to be fully elucidated, but it appears to be involved in multiple cellular and behavioral functions (Jordan and Xi, 2019). There is also evidence that AEA activates TRPV1 ion channels (van der Stelt et al., 2005), which have been shown to modulate habitual behavior (Shan et al., 2015). But also see (Gianessi et al., 2019), who recently reported that antagonism of TRPV1 does not influence habit formation. The GPR55 orphan receptor, which is thought to be activated by both eCBs and synthetic cannabinoids (Marichal-Cancino et al., 2017), was also reported to influence learning in a T-maze (Marichal-Cancino et al., 2016). Peroxisome proliferator-activated receptors (PPAR) are yet another target worth considering. PPARs are activated by various lipids, including eCBs (Iannotti and Vitale, 2021), and are thought to influence DA release (Melis et al., 2013a). In all, at least 12 different receptors are known to be activated by eCBs (Maccarrone, 2020), suggesting that the scope of mechanisms through which phytocannabinoids, synthetic cannabinoids, and eCBs regulate behavior are considerably more complex than our current conception.

### eCBs, DSI, and a Model of DA Release

A unique feature of eCB signaling is that these molecules are not stored in vesicles like classical neurotransmitters but are instead synthesized *de novo* and released from post-synaptic neurons in times of sustained neuronal activity (Freund et al., 2003; Castillo et al., 2012; Ohno-Shosaku and Kano, 2014). Heightened neural activity results in increased intracellular Ca^2+^ that leads to the activation of synthetic enzymes (DAGL, NAPE-PLD) responsible for the rapid synthesis of eCBs (Marsicano et al., 2003; Lu and Mackie, 2016). Following their release from the postsynaptic neuron into the synaptic cleft, eCBs retrogradely activate CB1s located on presynaptic terminals of both GABA and glutamate neurons (Wilson and Nicoll, 2002; Melis et al., 2004; Alger and Kim, 2011). Retrograde eCB modulation of GABA terminals can produce depolarization-induced suppression of inhibition (DSI), whereas retrograde eCB modulation of glutamate terminals can produce depolarization-induced suppression of excitation (DSE) (Fortin and Levine, 2007; Lange et al., 2017). In DSI, eCB activation of CB1s on GABA terminals is thought to produce a transient suppression of GABA release onto the postsynaptic neuron, thereby disinhibiting it. By contrast, during DSE, eCB activation of CB1s on glutamate terminals is thought to produce a transient suppression of glutamate release onto the postsynaptic neuron, thereby inhibiting it. While CB1 activation mediates both DSI and DSE, DSI is believed to be much more prominent than DSE due to differences in CB1 sensitivity between inhibitory and excitatory synapses (Ohno-Shosaku et al., 2002).

Cannabinoid receptor type 1-mediated DSI provides a model that might explain how phytocannabinoids, synthetic cannabinoids, and eCBs increase DA release from the midbrain. For a thorough description of how DSI is thought to modulate DA release, we refer the reader to a previously published review clarifying the mechanisms involved (Covey et al., 2017). In the midbrain, CB1s are thought to occur on GABAergic and glutamatergic terminals rather than on DA neurons (Julian et al., 2003; Mátyás et al., 2008). In the awake and behaving animal, midbrain DA neurons fire in one of two distinct patterns: tonic and phasic (Grace and Bunney, 1984; Grace, 1991; Grace et al., 2007). At rest, DA neurons are tonically active and exhibit steady pacemaker activity, firing at an average rate of 5 Hz. By contrast, DA neurons fire in phasic bursts of 10–20 Hz when an animal is presented with a motivationally salient stimulus (Grace et al., 2007). These phasic bursts are thought to give rise to high-concentration transient DA release events in the NAc that encode the value of motivationally salient stimuli and actuate goal seeking (Wise, 2004; Grace et al., 2007; Schultz et al., 2015; Stauffer et al., 2016). Burst firing of DA neurons also leads to eCB synthesis, retrograde signaling, and activation of CB1s on GABA and glutamate terminals (Szabo et al., 2002; Riegel and Lupica, 2004; Melis et al., 2013b; Wang et al., 2015). If DSI is more prevalent than DSE in the midbrain, the result would be disinhibition of DA neurons and the subsequent release of DA at terminal sites of the mesocorticolimbic and nigrostriatal DA pathways. In support of this model, a growing body of evidence using a multitude of techniques report that eCBs (Solinas et al., 2006; Oleson et al., 2012), THC (Chen et al., 1990, 1993; Diana et al., 1998; Gessa et al., 1998; Voruganti et al., 2001; Pistis et al., 2002; Bossong et al., 2009), and synthetic cannabinoids (Tanda et al., 1997; Diana et al., 1998; Gessa et al., 1998; Fadda et al., 2006; Oleson et al., 2014) increase striatal brain DA levels in both rodents (Gessa et al., 1998; Pistis et al., 2002; Fadda et al., 2006) and humans (Voruganti et al., 2001; Bossong et al., 2009, 2015). Using *in vitro* electrophysiology, Melis et al. (2013b) demonstrated that this DSI-induced disinhibition of DA release is principally mediated by 2AG activating CB1s. Indeed, several studies will be presented herein demonstrating that the eCB 2AG effectively modulates DA-associated behavior in a CB1 dependent manner. However, while this model may explain the effects of synthetic cannabinoids and eCBs on DA release presented within this review, we acknowledge that it is incomplete because it does not account for the role of CB2 or other receptor targets (e.g., PPARs) that likely modulate DA release as well.

### eCB Modulation of the Mesocorticolimbic System

The mesocorticolimbic DA system originates from DA neurons in the ventral tegmental area (VTA) that project to a variety of brain regions. Its most prominent target is the ventral portion of the striatum, or nucleus accumbens (NAc) (Morales and Margolis, 2017). While the VTA is primarily composed of DA neurons (∼60%), GABA (∼25%) and glutamate (∼15%) neurons also exist and are capable of modulating DA neural activity, mesocorticolimbic output, and behavior (Swanson, 1982; Morales and Root, 2014; Yoo et al., 2016). It is theorized that these neurons form subpopulations that then receive disproportionate afferent input from distinct brain structures (e.g., periaqueductal gray, lateral hypothalamus, raphe nuclei, rostromedial tegmental nucleus) to form dissociable microcircuits that may subserve unique behavioral functions (Lammel et al., 2014; Breton et al., 2019). Thus, in addition to disinhibiting DA release in the VTA, it is likely that eCBs also modulate DA-associated behavior by acting on distinct afferents that then synapse onto DA neurons. It is also worth considering eCB modulation of neural activity at terminal fields of the mesocorticolimbic system. Like other monoamines, DA functions as a relatively slow neuromodulator of fast glutamate- and GABA-mediated neurotransmission and, in the awake and behaving rat, the effect that DA exerts on postsynaptic potentials is greatly influenced by these converging inputs into a given terminal field (O’Donnell et al., 1999; Brady and O’Donnell, 2004). As a prominent mesocorticolimbic hub, the effect DA exerts in the NAc can therefore be influenced by eCB modulation of amygdalar, hippocampal, and cortical input into it. The neuromodulatory effects of DA in the NAc can also be influenced by co-release of GABA and glutamate from VTA DA neurons. Emerging evidence suggests that VTA DA neurons are capable of co-releasing GABA and glutamate in a manner that regulates motivational drive along with DA (Tritsch et al., 2012; Zhang et al., 2015; Yoo et al., 2016).

Dopamine signaling in the NAc is primarily mediated through D1- and D2-like receptors. D1 receptors generally exhibit low binding affinity for DA and preferentially couple to G_*s*_ protein subunits. D2 receptors generally exhibit high binding affinity for DA and preferential coupling to G_*i*_ or G_*o*_ protein subunits (Beaulieu and Gainetdinov, 2011). Both D1 and D2 DA receptors are expressed as heteroreceptors on dendritic spines of medium spiny GABA neurons (MSNs) within the NAc (Levey et al., 1993; Monory et al., 2007), though D2s are also expressed on presynaptic DA terminals where they function as autoreceptors to attenuate DA release (Bello et al., 2011; Budygin et al., 2017). Notably, CB1s form heterodimeric complex with D2s where colocalization exists, suggesting CB1 may interact with D2 autoreceptors to modulate DA release (Khan and Lee, 2014). It is also noteworthy that CB1s are expressed in a subpopulation of fast-spiking interneurons (FSI) within the NAc (Winters et al., 2012). Despite only composing 2–3% of striatal neurons, FSI are thought to powerfully orchestrate the activity of the more predominate MSNs and, possibly, control gamma frequency oscillations originating from this region (Tepper et al., 2010). Individual FSIs innervate hundreds of MSNs and, when excited, inhibit their collective output (Tepper et al., 2008). Within the NAc, eCBs are synthesized and released from MSNs following activation of either D1 (Shonesy et al., 2020) or D2 (Lerner and Kreitzer, 2012) receptors. Also noteworthy is recent evidence suggesting that tyrosine receptor kinase B activation augments intracellular calcium transients to promote eCB synthesis and spike-timing dependent plasticity in the striatum (Gangarossa et al., 2020). Upon release from MSNs, the eCBs then travel retrogradely before acting upon CB1s located on FSIs within the NAc (Mateo et al., 2017; Wright et al., 2017). Wright et al. (2017) recently used electrophysiology to demonstrate that the inhibitory control that CB1-expressing FSIs exert over MSNs is suppressed by eCB signaling. In addition, these authors (Wright et al., 2017) found that the CB1 expressing FSIs that synapse onto MSNs facilitate a long-term form of eCB-mediated synaptic plasticity (i.e., long-term depression, LTD) that might be important for learning and memory. While previous immunohistochemical studies suggested CB1s are also expressed on cholinergic interneurons within the striatum (Fusco et al., 2004), a more recent study using CB1 radioactive antisense riboprobes found no evidence of CB1 mRNA expression within cholinergic interneurons of the NAc (Mateo et al., 2017). This latter finding is particularly relevant for the current review because we primarily focus on transient DA release events in the NAc. And, it is now recognized that these transient release events can be promoted by either local cholinergic interneurons that activate acetylcholine receptors on adjacent DA terminals (Cachope et al., 2012) or by VTA DA cell activation, both of which are modulated by eCB signaling (Mateo et al., 2017). However, the lack of CB1s on cholinergic interneurons in the NAc suggests that eCBs modulate the influence local cholinergic interneurons exert over terminal dopamine release indirectly. In support of this notion, Mateo et al. (2017) recently reported that eCB modulation of cholinergic-induced terminal dopamine release results from CB1 activation on cortical glutamate afferents into the NAc. In addition to the aforementioned findings by (Wright et al., 2017), this latter observation is highly relevant for eCB- and DA-modulation of learning and memory. D2-dependent eCB-LTD has been verified in glutamatergic corticostriatal projections within the indirect pathway of the basal ganglia—a group of subcortical nuclei including the striatum that modulate behavioral action, procedural learning, and working memory (Lerner and Kreitzer, 2012; Simonyan, 2019). Thus, eCBs likely influence skill learning and memory by indirectly modulating terminal DA release and by gating FSI-control of MSN feedforward inhibition. These separate mechanisms—comprising eCB-mediated DSI/DSE within the VTA, eCB modulation of neural signaling with the NAc, and eCB modulation of afferent input into the NAc and VTA—may all converge to influence mesolimbic DA neurotransmission (Covey et al., 2017).

### A Synthetic Cannabinoid Dose-Dependently Increases DA Release and Tolerance Develops to This Effect Following Chronic Exposure

**FIGURE 1:**
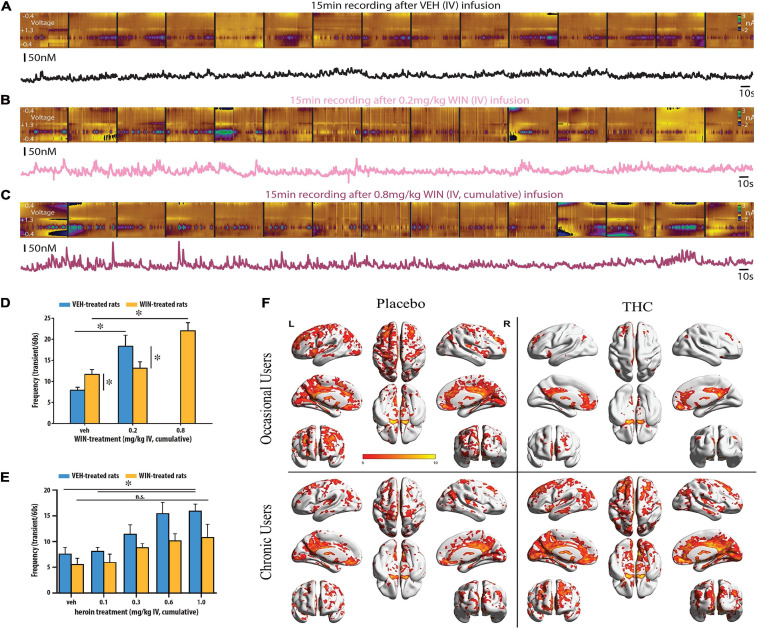
Cannabinoids increase the frequency and amplitude of DA transients. Illustrative recording session in which the synthetic cannabinoid WIN was administered to an awake and freely moving rat. Stitched color plots [voltammetric current (*z*-axis) × applied scan potential (*y*-axis) × time (*x*-axis)] are shown above corresponding DA concentration traces. Vehicle **(A)**, 0.2 mg/kg **(B),** and 0.8 mg/kg WIN **(C)** were administered in cumulative, ascending IV doses while FSCV measurements of DA release events occurred in the NAc shell in near real-time. Dose dependent increases in the frequency and amplitude of DA release events can be observed by the larger and more frequent green dots at a potential of +0.6 V in the color plots and the more frequent and pronounced transient peaks in the corresponding DA concentration traces. **(D)** WIN increased the frequency of DA release events but was less potent in chronically WIN-treated rats. A higher dose of WIN (0.8 vs. 0.2 mg/kg IV) was required to produce a significant increase in DA release vs. vehicle treated rats. **(E)** Heroin dose-dependently increased the frequency of DA release events but was less effective in chronically WIN-treated rats. In WIN-treated rats, heroin did not significantly increase the frequency of DA transients vs. vehicle at any dose tested. Republished from Gomez et al. (2020). **(F)** NAc–related functional connectivity in the left hemispheres. Shown are thresholded *Z*–score maps of functional connectivity for each group and each condition. Smoked THC reduced functional connectivity between the NAc and broad areas of the frontal, temporal, parietal and occipital lobes in occasional, but not chronic cannabis users. Republished from Gomez et al. (2020). ^∗^*p* < 0.05.

Abused drugs are theorized to exert their reinforcing effects by mimicking these endogenous patterns of DA release in the NAc that normally strengthen goal-directed behavior (Volkow and Morales, 2015; Volkow et al., 2017), and cannabinoids are no exception. We recently confirmed that the synthetic cannabinoid WIN increases accumbal transient DA release events in a dose-dependent manner and further investigated whether tolerance develops to this effect. We used fast-scan cyclic voltammetry (FSCV) to measure sub-second DA transients using NAc-implanted electrodes while treating awake and behaving rats with increasing, cumulative doses of intravenous (IV) WIN. As illustrated in Figures 1A–C, WIN dose-dependently increased both the frequency and amplitude of transient DA release events in the NAc shell (Gomez et al., 2020). We next wanted to assess whether chronic WIN exposure produces tolerance to the DA releasing effects of WIN. Although chronic treatment with synthetic or phytocannabinoids is known to produce tolerance to a tetrad of behavioral/physiological effects that is used to screen whether a drug functions as a cannabinoid (i.e., antinociception, catalepsy, hyopthermia, and hypomotility) (Little et al., 1988; Wiley and Martin, 2003; Hama and Sagen, 2009; Nealon et al., 2019)—it remains unclear whether tolerance develops to the rewarding/reinforcing and the DA releasing effects of cannabinoids. Because the degree of tolerance that develops to specific cannabimimetic effects varies as a result of CB1 desensitization occurring in a brain region-dependent manner (Breivogel et al., 1997; Whitlow et al., 2003), it is possible that midbrain CB1s show resistance to tolerance. Supporting this notion, Frau et al. (2019) found that prenatal exposure to THC produces a hyperDAergic rather than a hypoDAergic phenotype, Mavrikaki et al. (2010) found that chronic WIN exposure does not alter brain-reward thresholds, Hirvonen et al. (2012) found that CB1s are downregulated in cortical but not subcortical regions of cannabis smokers, and Wu and French (2000) found that chronic THC treatment does not influence its ability to induce burst firing in putative DA neurons.

To test whether tolerance develops to the DA-releasing effects of WIN, we treated rats with either vehicle or intravenous (IV) WIN using an escalating dosing regimen. To determine if this dosing regimen produced tolerance to standard cannabimimetic effects, we first tested the consequences of it using the tetrad test. As expected, we found that WIN-treated rats displayed a rightward shift in the dose-response relationship (0.002–0.8 mg/kg IV) across all behavioral/physiological measures when compared to vehicle-treated controls. We then used FSCV to investigate whether the same pharmacological history produced tolerance to the DA releasing effects of WIN and cross-tolerance to the DA releasing effects of heroin. We additionally characterized whether this dosing regimen produces cross-tolerance to the DA releasing effects of heroin because Cadoni et al. (2008) observed this effect using microdialysis. In addition, synthetic cannabinoids/phytocannabinoids and opioids are well known to produce cross-tolerance to several shared neurobehavioral effects (Hine, 1985; Thorat and Bhargava, 1994; Manzanares et al., 1999; Vigano et al., 2005; Gerak et al., 2015). We found that after chronic WIN exposure, both WIN (Figure 1D) and heroin (Figure 1E) were less effective at increasing the frequency of DA release events in the NAc shell of adult male rats. If DA is important for drug reward (Di Chiara et al., 2004) or to motivate drug seeking (Volkow et al., 2017) as is currently theorized, a diminished ability to evoke DA release could promote the use of larger quantities and more potent doses. These data support a recent PET imaging study demonstrating that cannabis-dependent patients show a deficit in striatal DA release after the investigators controlled for several comorbidities that may have influenced previous imaging studies (van de Giessen et al., 2017). In another noteworthy imaging study, Mason et al. (2021) used resting-state functional magnetic resonance imaging (fMRI) to determine functional connectivity between the NAc and other brain regions of interest in occasional and chronic cannabis users. Both groups received placebo and 300-μg/kg THC on separate days. In occasional users, THC produced a marked reduction in functional connectivity between the NAc and broad areas of the frontal, temporal, parietal and occipital lobes (Figure 1F)—a pattern the authors note is typical of increased DA neurotransmission. In chronic users, THC did not produce changes in functional connectivity associated with the NAc (Figure 1F). The occasional, but not chronic cannabis users, also reported increases in subjective high and showed impairments in a sustained attention task. From these observations, the authors conclude that excessive cannabis use may result in neuroadaptations in accumbal circuitry that reduce the neurobiological and behavioral response to acute cannabis impairment.

However, further studies are necessary to compare how synthetic cannabinoids, eCBs, and phytocannabinoids produce tolerance, whether each produces tolerance to the DA releasing effects of a CB1 agonist, and whether these effects vary with age, sex, or species. It is possible that a synthetic aminoalkylindole cannabinoid like WIN produce distinct effects on molecular, cellular, and/or behavioral tolerance in comparison to a phytocannabinoid like THC. Two complimentary molecular mechanism are thought to contribute to CB1 desensitization and downregulation (Nguyen et al., 2012; Nealon et al., 2019). One involves the recruitment of beta-arrestin2 to GRK-phosphorylated CB1s (Jin et al., 1999; Nguyen et al., 2012). The other is a distinct JNK-mediated form of molecular tolerance that appears to occur in an agonist specific manner (Nealon et al., 2019). Of note, it was recently reported that disrupting JNK signaling prevents several forms of behavioral tolerance induced by THC, but not by WIN (Henderson-Redmond et al., 2020). Thus, future studies are needed to determine how different cannabinoid ligands produce tolerance to distinct behavioral/physiological effects.

## Cannabinoids and Motivated Action Under a Variety of Reinforcement Contingencies

### Response Reinforcement and Schedule-Controlled Behavior

**FIGURE 2:**
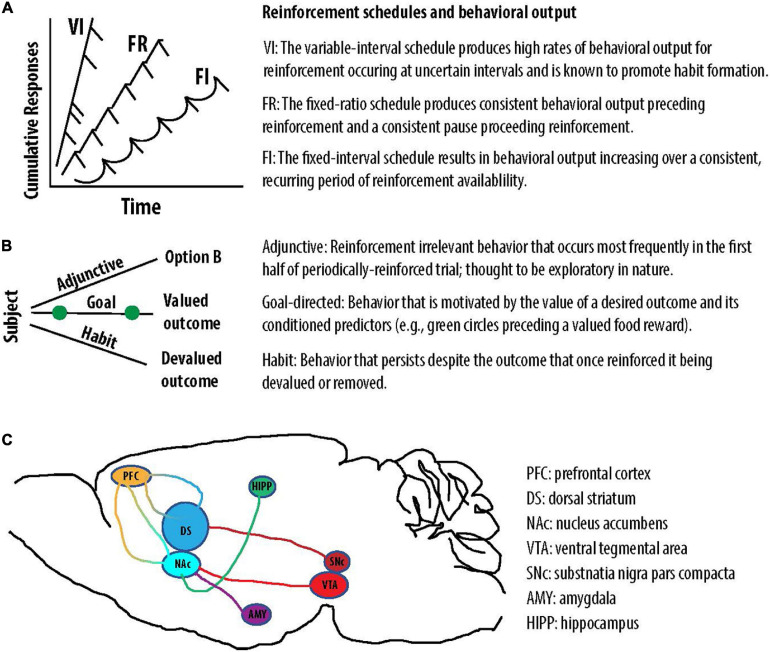
Reinforcement schedules engender distinct behaviors and a depiction of two DA pathways. **(A)** The VI, FR, and FI schedules produce unique patterns of reinforced behavior. **(B)** The contingencies of reinforcement can produce adjunctive, goal-directed, or habitual behavior. **(C)** Illustrative projections associated with the nigrostriatal (dark red) and mesocorticolimbic (light red) DA pathways. DA in the NAc is thought to modulate converging input from brain regions including the amygdala (AMY), hippocampus (HIPP), and prefrontal cortex (PFC). Cortico-striatal loops are depicted in multicolor (orange-blue).

While many different behavioral approaches exist to study the effects of cannabinoids on behavior, this review will primarily focus on response reinforcement and operant behavior. Response reinforcement was first described by Thorndike (1927) as a law of effect—meaning that responses following a satisfying connection act upon it to alter its strength. Concepts associated with the law of effect were further explored in great detail following Skinner’s inventions of the operant conditioning chamber and the cumulative recorder (Ferster and Skinner, 1957). The operant conditioning chamber allows experimenters to measure repeatable responses in the face of changing conditions. The cumulative recorder produced a graphical record of the animal’s responses, allowing experimenters to study how changing conditions influence the probability of a response. Using this new technology, Ferster and Skinner (1957) reported that the pattern of responses can be greatly influenced by the reinforcement schedule. In the operant context, schedules can be thought of as the rules under which reinforcement is made available, or the contingencies of reinforcement. The observation that reinforcement schedules powerfully modify operant behavior had profound implications for our understanding of the phylogeny of behavior and neurobiology. In an evolutionary context, it is likely that patterns of behaviors were neurobiologically stamped-in when they maximized the receipt of an advantageous outcome (e.g., food) in the face of changing environmental conditions (e.g., the periodic availability of food). Because the environment changes in recurring patterns, it would therefore be advantageous for the brain to produce complex patterns of behavior that adapt to the environmental rules governing reinforcement (Skinner, 1966). In the context of cannabinoid effects on the brain and behavior, it is equally important to recognize that a drug or neurochemical can produce unique effects on operant behavior under different schedules of reinforcement. This phenomenon was first described by Peter Dews, who used an operant conditioning chamber and cumulative recorder to demonstrate that injecting pigeons with the same dose of pentobarbital increased responding for food under a fixed-ratio scheduled but decreased responding for food under a fixed-interval schedule (Dews, 1955). Under a fixed ratio (FR) schedule, behavior is reinforced after the animal responds a pre-defined number of times. This contingency of reinforcement produces a bimodal step-like pattern in which the animal is either responding at a constant rate or at zero (Ferster and Skinner, 1957) (Figure 2A). Under a fixed interval (FI) schedule, behavior is reinforced after the animal responds after a pre-defined period of time. This contingency of reinforcement produces a scalloped-like pattern of responding (Dews, 1978) (Figure 2A). Because this review will focus on the interaction between cannabinoids and DA signaling in particular, it is also worth noting that DA pharmacology is well known to produce divergent behavioral effects under these two schedules of reinforcement. Equivalent doses of the DA releasers amphetamine and methamphetamine (Cho, 1990; Jones et al., 1998) both decrease response rate under an FR1 schedule and increase response rate under a FI schedule (Dews, 1958; McKearney and Barrett, 1978).

### DA Value Signals in Reinforcement and Goal-Directed Action

**FIGURE 3:**
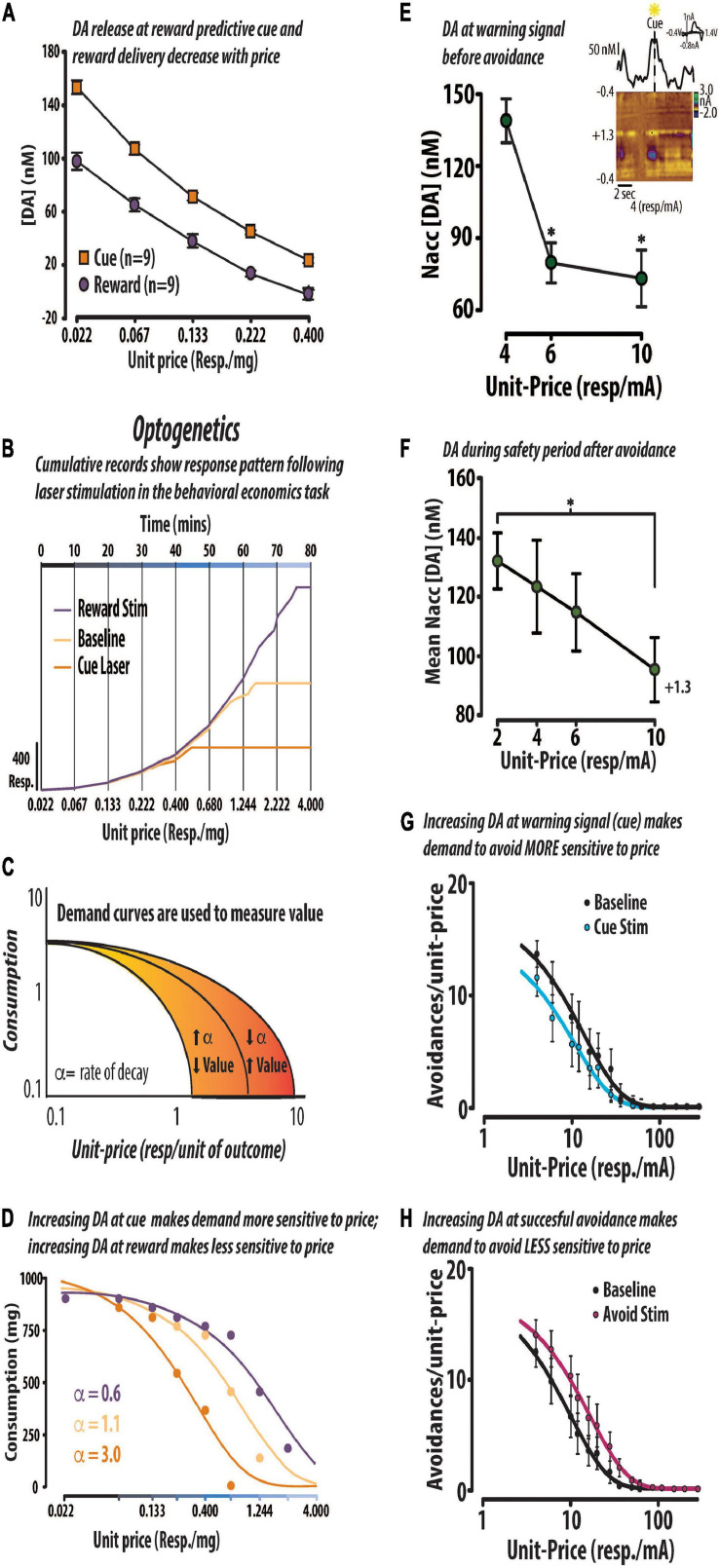
DA value signals encode price and modify the maximal price rats will pay for positive or negative reinforcement. *Positive reinforcement:* DA (DA) concentration (mean ± SEM) evoked by a reward predictive cue and delivery of a 45 mg sugar pellet decreased across the first five prices in a within-session behavioral economics-based task. In this task, the unit-price (responses/mg sugar) increased across fixed epochs of time **(A)**. Optogenetic stimulation alters price sensitivity in a representative rat. Cumulative response records from one animal responding in the behavioral economic task under baseline conditions (light orange), and those in which DA release is amplified at cue presentation (dark orange) and at reward delivery (purple) **(B)**. Changes in value were assessed using demand curves which measure changes in consumption in response to changes in unit-price. We formally extracted a dependent measure of value (i.e., α) which, represents the rate at which demand curve decay. Demand decays at a faster rate when the animal becomes more sensitive to price. As the animal is willing to pay less for the commodity, we would interpret the resulting increase in α as a decrease in value **(C)**. The same data from the cumulative records in panel **(B)** are replotted in the form of demand curves to illustrate the optogenetic-induced shifts in value **(D)**. *Negative reinforcement:* The concentration of DA evoked by a warning signal that predicted the opportunity to avoid decreased with the price to avoid. Inset: Representative avoidance trial shows that DA concentration began increasing in anticipation of warning signal presentation **(E)**. The concentration of DA release events during the safety period decreased with price in trials in which the rat successfully avoided electrical foot shock **(F)**. Optogenetic activation of VTA DA neurons at the warning signal made animals more sensitive to price, consistent with a negative reward prediction error **(G)**. In contrast, optically stimulating DA neurons at successful avoidance made animals less sensitive to price, consistent with a positive reward prediction error **(H)**. Republished from Schelp et al. (2017) and Pultorak et al. (2018). ^∗^*p* < 0.05.

In the awake and behaving animal, midbrain DA neurons fire in phasic bursts (>20 Hz) under a variety of conditions (Redgrave et al., 2016; Sharpe and Schoenbaum, 2018), including the presentation of rewarding stimuli (Stauffer et al., 2016). These phasic bursts of neural activity contribute to transient DA release events in the primary terminal field of the mesolimbic pathway, the nucleus accumbens (Dreyer et al., 2010). Currently, it is thought that transient DA signals within this brain region encode value as positive or negative reward prediction errors. In support of this theory, a series of *in vivo* electrophysiology studies demonstrated that phasic bursts of DA neural activity respond to gambles that guide economic decision making and integrate various factors that underlie value representations to influence choice (Lak et al., 2014, 2016; Stauffer et al., 2014, 2016). We recently tested the notion that transient DA value signals represent value and influence valuation during both positive and negative reinforcement (Figure 3) (Schelp et al., 2017; Pultorak et al., 2018; Oleson and Roberts, 2019). Positive reinforcement refers to an increase in behavior to receive an outcome (e.g., appetitive sugar pellet); negative reinforcement refers to an increase in behavior to avoid an outcome (e.g., electrical footshock). Using FSCV, we first demonstrated that the concentration of transient DA release events evoked by an appetitive sugar pellet or its conditioned predictor decreased with the price required to obtain it (Figure 3A) (Schelp et al., 2017). DA release events and behavioral output were measured as rats responded in a within-session behavioral economics-based operant task. In this task, the unit-price (responses/mg sugar) to obtain reinforcement increased in fixed epochs over the course of each session. As illustrated by the representative cumulative response records in Figure 3B, under these response contingencies lever pressing increases across the fixed epochs (as price increases) until a maximal price is reached at which the animal is no longer willing to pay the required opportunity cost to obtain reinforcement. We then used optogenetics to augment DA release and found that increasing DA release at the reward predictive stimulus rendered animals more sensitive to price and decreased DA concentration at reward delivery, consistent with a negative reward prediction error (Schelp et al., 2017). Optogenetics is a neuroscientific technique that allows the experimenter to transiently turn on/off a neural population of interest by activating genetically introduced light sensitive ion channels (i.e., opsins) with a laser (Vlasov et al., 2018). In comparison to this animal’s baseline cumulative record (light orange line) increasing DA release at reward delivery (purple line) resulted in the animal paying a higher price to continue seeking sugar; whereas, increasing DA release at the reward predictive cue (dark orange line) resulted in the animal giving up at a lower price (Figure 3B). We then converted the behavioral data into demand curves by calculating total sugar in each epoch and plotting it against the corresponding unit-price. Demand curves are a common tool used by economists to measure changes in valuation. If demand becomes more sensitive to price it is said to be more elastic, suggesting diminished value; if demand becomes less sensitive to price it is said to be more inelastic, suggesting enhanced value (Figure 3C). Replotting the same data from the aforementioned cumulative records revealed that enhancing DA release at cue presentation made demand for sugar more elastic, while enhancing DA release at reward delivery made demand for sugar more inelastic (Figure 3D). From these observations, we infer that valuation of the sugar pellet was decreased when the DA value signal was amplified at the reward predictive cue because the animal perceived that they received less than expected upon receiving the standard 45 mg sugar pellet. By contrast, an amplified DA value signal at the receipt of the 45 mg sugar pellet following a standard prediction might suggest to the animal that they received a better bargain than expected. Similar observations were observed during operant behavior maintained by the avoidance of electrical footshock (Wenzel et al., 2015; Pultorak et al., 2018). The concentration of DA release events—evoked by both a warning signal predicting the delivery of electrical footshock and by the successful avoidance of footshock—decreased with the price required to avoid it (Figures 3E,F) (Pultorak et al., 2018). Furthermore, optogenetically increasing DA release at the warning signal made the demand to avoid more sensitive to price (Figure 3G) whereas, increasing DA release at successful avoidance made demand for avoidance less sensitive to price (Figure 3H) (Pultorak et al., 2018). Taken together, these findings support the notion that transient DA signals can represent subjective value during both positive and negative reinforcement and causally modify reinforcement processes.

### ECB Signaling Modulates DA Value Signals and Reinforcement Under a Fixed Ratio Schedule

**FIGURE 4:**
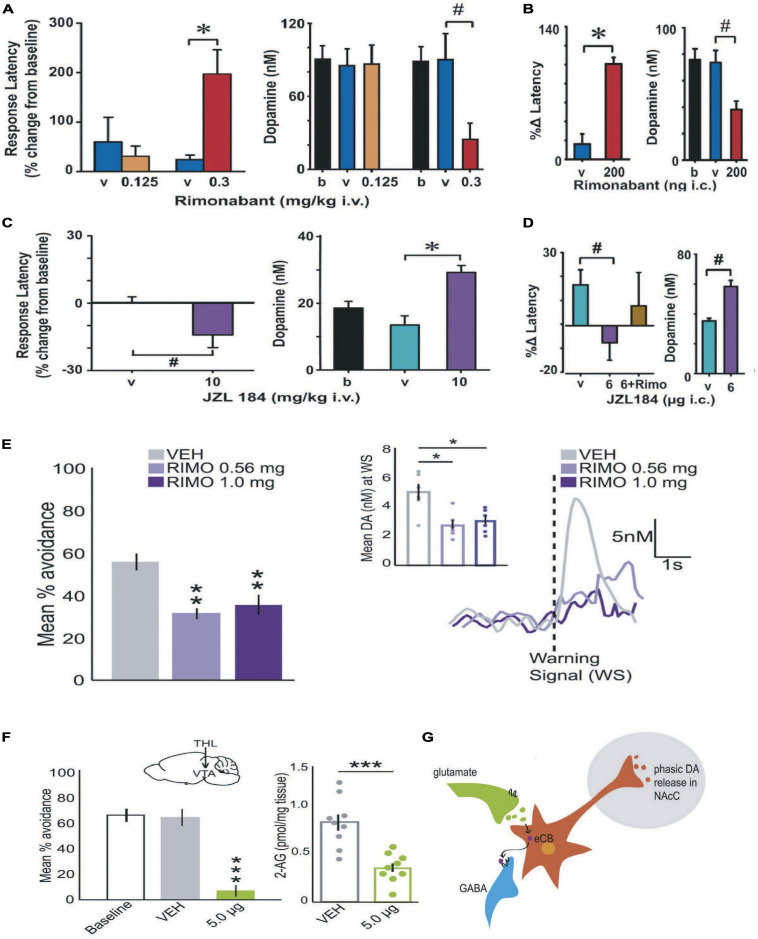
Cannabinoids modulate DA value signals during positive and negative reinforcement maintained under an FR schedule. *Positive reinforcement:* Systemically treating (intravenous; IV) rats with the cannabinoid receptor antagonist rimonabant increased the latency to respond for brain stimulation reward and decreased the concentration of cue-evoked DA value signals **(A).** Intrategmental infusions (IC) of rimonabant recapitulated these effects on reward seeking and DA release, demonstrating that eCB modulation of DA neural activity in the VTA is alone sufficient to modulate DA release and positive reinforcement **(B)**. Systemically increasing 2AG levels by pre-treating rats with JZL184 (IV) reduced the latency to respond for brain stimulation reward and increased the concentration of cue-evoked DA value signals **(C)**. Intrategmental infusions (IC) of JZL184 recapitulated these effects, suggesting that the action of 2AG in the VTA is alone sufficient to modulate DA release and positive reinforcement **(D)**. *Negative reinforcement:* Systemic rimonabant administration (IV) reduced the number of successful avoidance responses and the concentration of DA evoked by the warning signal **(E)**. Inhibiting DAGL-induced synthesis of 2AG by infusing THL into the VTA decreased avoidance and reduced 2AG tissue content in the VTA **(F)**. Taken together, these observations generally support a DSI-model of 2AG-modulation of DA value signals **(G)** during positive and negative reinforcement maintained under an FR schedule. Republished from Oleson et al. (2012) and Wenzel et al. (2018). # < 0.05; **p < 0.001; ***p ≤ 0.001.

Given the well-established role DA value signals play in reinforcement and motivating action (Schultz et al., 2015), we next began to question whether the brain’s endogenous cannabinoid system capably modulates transient DA release events during goal-directed behavior. Inspiration for this research question originated from early psychopharmacological studies. It was reported that disrupting eCB signaling by treating rats with CB1 antagonists reduced food seeking (Ward and Dykstra, 2005) and generally diminished the effects that conditioned stimuli exert over goal-directed behavior (Stiglick and Kalant, 1982; Le Foll and Goldberg, 2005; Ward et al., 2007). Thus, we first assessed whether treating rats with a CB1 antagonist reduced conditioned DA release events during positive reinforcement. Two reinforcers were assessed: brain stimulation reward and appetitive food. In the case of brain stimulation reward, rats responded for electrical currents delivered to the origin of the mesolimbic DA pathway—the VTA, under a FR1 schedule of reinforcement. The availability of reinforcement was signaled to the rat by a cue light placed above the lever, which began to function as a conditioned stimulus. Under these conditions, the concentration of DA value signals evoked by the cue light increased across trials as reinforcement was strengthened (Day et al., 2007; Oleson et al., 2012). Once DA value signals were determined to be stable, we intravenously treated rats with vehicle and then a CB1 antagonist (SR141716; AKA, rimonabant). In comparison to vehicle, systemic administration of the CB1 antagonist rimonabant significantly decreased the DA value signal while concurrently delaying reinforced responding (Figure 4A). Identical trends were found when we measured DA value signals while rats responded for 45 mg sugar pellets under a FR1 reinforcement schedule, demonstrating the reliability of these results during positive reinforcement (Oleson et al., 2012). And identical trends were found when we infused rimonabant directly into the VTA during brain stimulation reward (Figure 4B), suggesting that local eCB modulation of DA release in the midbrain is alone sufficient to modulate DA value signals and reward seeking. To assess whether increasing eCB signaling facilitates positive reinforcement, we then treated rats with an enzymatic inhibitor that prevents metabolic degradation. We focused on MAGL inhibitors because FAAH inhibitors failed to influence reinforcement in our initial studies (Oleson et al., 2012, 2014) and 2AG is thought to be the principle eCB that augments DA release by activating CB1s on GABA terminals (Covey et al., 2017). We replicated our aforementioned approach by intravenously administered the MAGL inhibitor JZL184 while rats responded for brain stimulation reward under a FR1 schedule during ongoing FSCV measurements of DA value signals. In contrast to rimonabant, intravenous JZL184 amplified DA value signals while concurrently reducing response latencies (Figure 4C). The same trends were observed when JZL184 was infused directly in the VTA (Figure 4D). Using a new and improved iteration of MAGL inhibitor called MJN110, the Bass lab recently replicated these findings by demonstrating increasing 2AG facilitates cue-motivated reward seeking (Feja et al., 2020). To determine whether eCBs modulate DA value signals during negative reinforcement we also assessed whether systemic administration of a CB1 antagonist influences DA value signals during avoidance. Using a signaled active avoidance operant approach, we treated rats with the CB1 antagonist rimonabant while conducting FSCV. Avoidance was maintained under a FR1 schedule. A warning signal was provided 2s prior to the occurrence of electrical foot shock by illuminating a cue light placed directly above the lever. In comparison to vehicle treatment, intravenous rimonabant significantly decreased DA release time-locked to the warning signal while concurrently decreasing avoidance (Figure 4E) (Wenzel et al., 2018). We next sought to assess whether 2AG manipulations specifically modify the influence of DA value signals on negative reinforcement. To do this we administered microinfusions of either vehicle or tetrahydrolipstatin (THL) into the VTA of rats. THL is a potent inhibitor of the synthetic enzyme responsible for generating 2AG, DAGL (Ortar et al., 2008). As predicted, intrategmental THL significantly reduced avoidance and 2AG tissue content in comparison to vehicle treated rats (Figure 4F) (Wenzel et al., 2018). Finally, we used optogenetics to stimulate DA neurons during avoidance and found that restoring DA value signals in the presence of THL was sufficient to rescue avoidance (Wenzel et al., 2018). Together, these findings suggest that the eCB 2AG facilitates cue-motivated action by amplifying DA value signals originating from the VTA (Figure 4G) (Oleson and Cheer, 2014; Covey et al., 2017; Wenzel et al., 2018; Peters et al., 2021). These 2AG-modulated patterns of DA release and behavior are apparent during both positive and negative reinforcement when a conditioned stimulus signals the availability of a goal-directed outcome and reinforcement is available under a FR schedule.

### Increasing Cannabinoids Amplifies Temporally Engendered Patterns of DA Release and Accelerates Responding Under a Fixed Interval Schedule of Reinforcement

**FIGURE 5:**
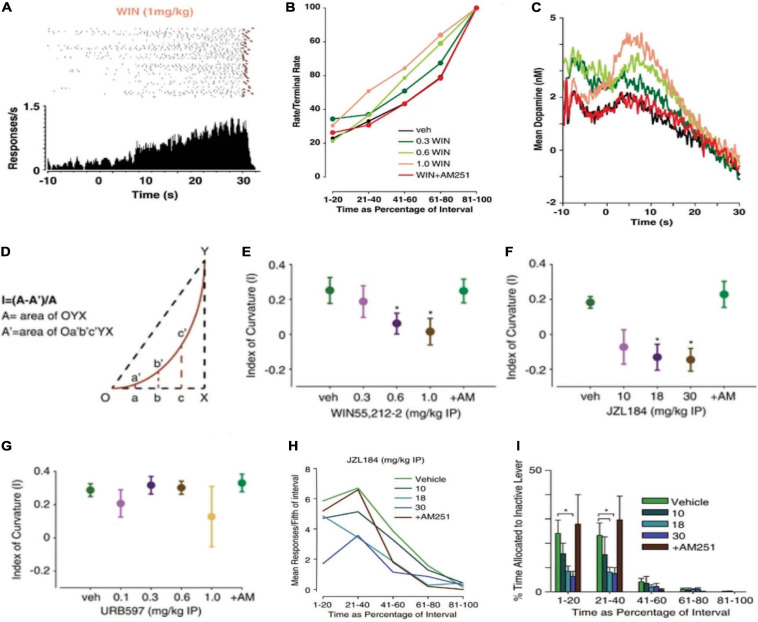
Cannabinoids modulate a temporally engendered pattern DA release during reinforcement maintained under an FI schedule and adjunctive behavior. An illustrative cumulative response pattern (top: raster plots; bottom: corresponding peri-event histograms) of a WIN-treated mouse responding for food reinforcement under a FI schedule. The pattern of lever pressing lawfully increases in the FI task to form a scalloped response pattern. The raster plot shows responses (black ticks) preceding food reinforcement (red triangle) across the 30 s interval. All trials are shown in chronological order as they occurred in a representative experimental session. The peri-event histogram shows the summation of responding under each corresponding raster plot. **(A)**. WIN 55,212-2 accelerated the timing of scallop response pattern in a dose- and CB1-dependent manner. Mean behavioral response patterns following cannabinoid administration are plotted as a function of the interval duration **(B)**. WIN amplified a temporally engendered pattern of DA release in a dose- and CB1-dependent manner. Mean DA concentration traces for each drug treatment conditions are plotted as a function of the interval **(C)**. Cannabinoid-induced changes in interval timing were quantified by assessing the index of curvature—a computational measure of the extent and direction of change in the temporal response pattern produced by the FI schedule **(D)**. WIN produced a negative index of curvature, suggesting an acceleration of timing behavior **(E)**. Increasing 2AG with JZL184, but not increasing anandamide with URB597, accelerated interval timing **(F,G)**. eCB-induced changes in reinforcement irrelevant or adjunctive behavior were assessed by quantifying responses on an inactive lever. Mean responses on the inactive lever initially increase before declining through the interval **(H)**. JZL184 significantly decreased the percentage of time spent responding on the inactive lever, suggesting that adjunctive behavior was reduced by elevating 2AG levels **(I)**. These data show that cannabinoids module periodically reinforced behavior and DA release under an FI schedule and, might suggest that a delicate balance of 2AG and DA release are necessary to produce the sweet-spot of intermittency that produces adjunctive behavior. Reproduced from Oleson et al. (2014).

Whereas FR schedules engender a bimodal response pattern consisting of recurring response-pause successions, the FI schedule engenders a scalloped response pattern. Rather than receiving reinforcement after meeting a fixed response requirement, on a FI schedule, reinforcement occurs at the end of a defined period of time. The lever does not retract during the interval, allowing the experimenter to observe the emergence of a scalloped temporal response pattern using a cumulative response recorder (Figure 5A). The scalloped response pattern results from the animal’s lever pressing accelerating across the interval until a maximum terminal rate is reached at the interval terminus (Ferster and Skinner, 1957). In addition to engendering a unique pattern of behavior relative to the FR schedule, the FI schedule also produces a unique pattern of accumbal DA release. As illustrated in Figure 5C, a first peak of DA release can be observed when reinforcement (an appetitive food pellet) is delivered. After a brief pause in release, DA concentration then begins to rise with the onset of the FI before gradually decaying over its duration (Oleson and Cheer, 2014; Oleson et al., 2014). As DA concentration is inversely related to local response rate, we infer that under the contingencies of a FI schedule, DA concentration represents the primary interoceptive cue driving reinforcement: time (Oleson et al., 2014; Everett et al., 2020). To investigate how cannabinoids alter both the patterns of behavior and DA release engendered by the FI schedule, we applied FSCV while treating mice with the cannabinoid agonist WIN as they responded for appetitive food pellets (Oleson et al., 2014). To analyze how responding changed across the interval, we first calculated rate/terminal rate values by dividing the local response rate into five fixed epochs and then dividing each by the terminal rate (i.e., the maximal local response rate in the final epoch). We found that WIN accelerated local response rates across the interval in a dose- and CB1-dependent manner (Figure 5B). Similarly, WIN dose-dependently increased DA concentration across the duration of the interval in a CB1-dependent manner (Figure 5C). We also performed a more refined behavioral analysis by assessing the index of curvature of individual scalloped response patterns (Figure 5D) (Fry et al., 1960; Narayanan et al., 2012). Using the index of curvature analysis, a negative index of curvature is detected when the animal’s scalloped response pattern accelerates prematurely; thereby suggesting that timing behavior is accelerated. By contrast, a slower acceleration of responding across the interval produces a positive index of curvature, suggesting that timing behavior is slowed (Fry et al., 1960; Narayanan et al., 2012). This additional analysis confirmed that WIN accelerated the timing of reinforced responding under a FI schedule while concurrently accelerating the temporally engendered pattern of DA release (Figure 5E). We then treated mice with enzymatic inhibitors to investigate whether specifically increasing the eCBs 2AG or anandamide modulate the scalloped response pattern observed during fixed interval reinforcement. We found that systemic treatment with the MAGL inhibitor JZL184, but not the FAAH inhibitor URB597 accelerated the temporal response pattern similarly to WIN (cf. Figures 5F,G,H vs. E,B) (Oleson et al., 2014). These data suggest that the eCB 2AG modulates goal-directed action under a variety of contingencies, including periodically reinforced behavior.

## Endocannabinoids, Exploratory/Adjunctive Behavior, and Habits

### Endocannabinoids and Exploratory/Adjunctive Behavior From an Ethological Perspective

We next consider these observations from a phylogenetic and ethological perspective. If cannabinoids amplify patterns of DA release and accelerate timing behavior under conditions of fixed periodic reinforcement, it is possible that they contribute to motivational switching in response to changing environmental conditions. After waiting a lengthy period of time for a primary food source, it may become advantageous to switch from seeking the desired option to foraging for alternative options. In the operant chamber, these foraging-like actions can be noted as reinforcement-irrelevant, or adjunctive behaviors (Falk, 1971, 1977). One proposed way to quantify adjunctive behavior in the operant chamber is the measure responding on a secondary inactive lever (Killeen and Fetterman, 1988). To assess whether cannabinoids influence adjunctive behavior, we reanalyzed the FI data and found that increasing 2AG using the MAGL inhibitor JZL184 (Figure 5I) or antagonizing CB1 with AM251 (Oleson et al., 2014) significantly reduced inactive lever presses. We interpret these findings to suggest that a basal eCB tone and a moderate concentration of accumbal DA provide the sweet spot of intermittency necessary to switch an animal’s incentive to obtain a primary goal (e.g., food) to the pursuit of alternative options (e.g., foraging for an alternative food source) (Oleson et al., 2014). Additionally, sudden increases or decreases in eCB signaling can lead to perseverative goal seeking. In agreement with this supposition, cannabinoids have been reported to promote perseverative action and infiexibility (Hill et al., 2006; Jiao et al., 2011).

### The Variable Interval Schedule and Habit Formation

Recent studies utilizing the variable interval (VI) schedule demonstrate that eCBs are critically involved in habit formation. As previously described, when reinforcement is delivered in fixed intervals, the animal learns to time the interval and accelerate their responding toward its culmination. By contrast, under a VI schedule, responding is reinforced after a random period of time has elapsed since the first response. Under these conditions, the cumulative response pattern is maintained at a high, constant rate—presumably because the animal is uncertain about the time of reward availability (Ferster and Skinner, 1957) (Figure 2A). In comparison to ratio schedules or the FI schedule, the VI schedule is known to produce habitual behavior (DeRusso et al., 2010). To determine if a behavior is habitual rather than goal-directed, experimenters determine if the instrumental action is driven by a valued outcome or devoid of its consequences (Figure 2B). To characterize and parse the purpose of action, Adams and Dickinson developed what is known as the devaluation test (Adams and Dickinson, 1981). After training an animal to respond for what was originally a valued outcome, the outcome is then devalued. In the case of food-maintained responding, the animal is either over-fed or subjected to food poisoning. If the animal’s responding is significantly affected by devaluation, it is inferred that action is still directed toward a valued goal; however, if the animal’s responding is insensitive to devaluation, it is inferred that action has become habitual. In the latter scenario, the habitual behavior is believed to be unresponsive to changes in outcome value and the contingency between action and outcome (Dickinson and Balleine, 1994).

### eCBs May Be Involved in Habit Formation

**FIGURE 6:**
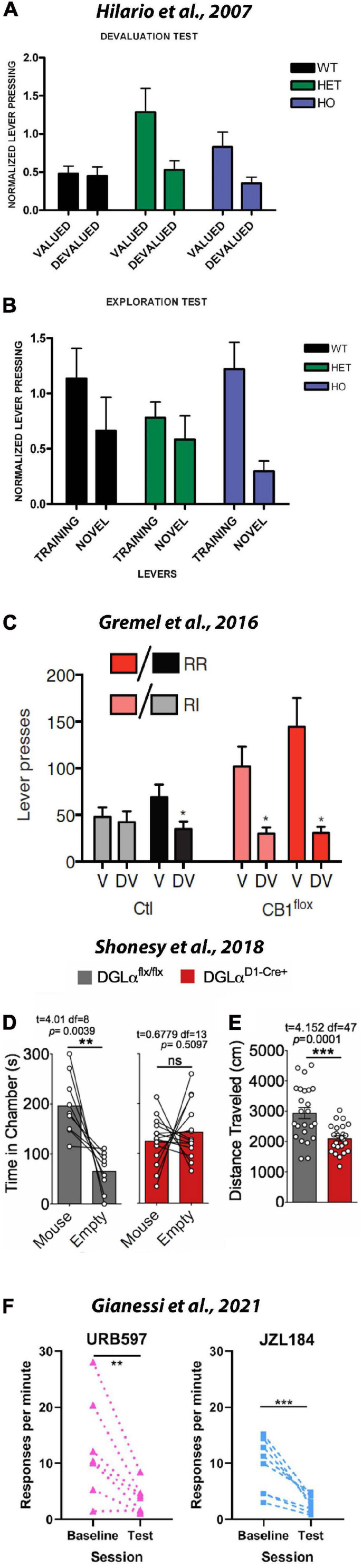
CB1s are necessary for habit formation and adjunctive/exploratory behavior. To investigate the role of eCBs in habit formation and adjunctive behavior WT, CB1^+/–^, and CB1^–/–^ were trained on a variable interval schedule and then tested in devaluation and exploration tests. **(A)** Normalized lever pressing during the valued versus the devalued condition for WT, CB1^+/–^, and CB1^–/–^ mice. CB1 mutants showed sensitivity to sensory-specific satiety, suggesting that their actions were goal-directed rather than habitual. These data suggest that the CB1 and eCBs are necessary for habit formation. **(B)** Lever pressing (normalized) on the training lever versus a novel lever in WT, CB1^+/–^, and CB1^–/–^ mice. Relative to other groups, CB1^–/–^ mice responded less on the novel lever, suggesting that the CB1 and eCBs may be involved in adjunctive behavior. (Republished from Hilário et al., 2007). **(C)** Graph shows responses in the valued (V) and devalued (DV) states in RI and RR training contexts. RR, random ratio (aka FR); RI, random interval (aka VI). During outcome devaluation procedures, control mice showed reduced lever pressing in the devalued state in the RR context but not the RI context. However, mice that lacked CB1s on OFC projection neurons into the striatum responded less in the devalued state under both RR and RI conditions. These data suggest that CB1s in cortical-striatal loops are necessary for habit formation (Republished from Gremel et al., 2016). Mice lacking the enzyme for the synthesis of 2AG from D1 MSNs (D1-Cre+) showed decreased exploration of a novel conspecific **(D)** and a novel environment **(E)**. These data suggest that 2AG in the striatum plays an important role during adjunctive behavior (Republished from Shonesy et al., 2018). **(F)** Surprisingly, blocking metabolism of AEA with URB597 and 2AG with JZL184 disrupted rather than promote habit formation. These latter findings might suggest that AEA and 2AG are not important in habit formation or that non-specific behavioral effects (e.g., increased motivation for food) can confound tests of habitual behavior. **p < 0.01; ***p ≤ 0.001.

Growing evidence suggests that eCB signaling is crucial for habit formation, although the precise roles each eCB play in habit formation and whether these roles differ at distinct loci in the brain remains to be determined. Hilário et al. (2007) first demonstrated a role for eCBs in habit formation. First, these authors confirmed that a history of responding under a VI-reinforcement schedule is particularly suited for establishing habitual responding. After providing mice with a history of responding for a sugar solution under either a VI schedule or a variable-ratio (i.e., VR) schedule, they were tested in a devaluation test. In this test all mice were given access to either a sugar solution (i.e., reinforcer from operant training) or standard chow (home cage food) for 1hr preceding an extinction session. During the extinction session, mice were given access to the sugar-paired lever used in operant training; however, no scheduled consequences occurred when it was pressed. As predicted, they found that a history of responding under VI schedule, but not the VR schedule, resulted in sugar-sated mice persevering in their responses on the sugar-paired lever. The authors also conducted a separate exploration test in which mice were given access to the previously active lever and a novel level. They found that in comparison to mice with a history under the VR schedule, mice with a history of responding under the VI schedule were more likely to engage with it. To test the effects of eCB signaling, the authors replicated their experimental approach using CB1 mutant mice and their wild-type littermates. After a history of responding for sugar under a VI schedule, wild-type (WT), heterozygous *CB*^+/–^ (HET) and homozygous *CB*^+/–^ (HO) mice were given access to the regularly active lever in either a sugar-sated or non-sated state. As shown in Figure 6A, devaluing the sugar solution failed to affect responding on the previously sugar-paired lever. This finding supports the notion that a history of responding under the VI schedule produces habitual responding. However, both the HET and HO groups CB1 mutant mice showed sensitivity to sugar devaluation. As evidenced by the green and blue bars, providing *ad libitum* access to the sugar solution before the devaluation test resulted in both CB1 mutant groups responding less in the sated state, suggesting that habit formation is impaired in CB1 mutant mice. As illustrated in Figure 6B, in the exploration test they found that HO mice, but not HET or WT mice, failed to explore the novel level. Taken together, these data suggest that CB1 signaling may play an important role in habit formation and exploring novel options. These data are in agreement with our aforementioned finding that pretreating mice with AM251 reduced inactive lever responses; although, it remains unclear why increasing 2AG levels with JZL184 also reduced adjunctive, or exploratory behavior. One likely possibility is that systemically increasing 2AG produces an array of physiological and behavioral effects at different levels of distinct neural networks. By harnessing recent technical advances, investigators are beginning to target specific cellular populations and neural circuits responsible for habit formation, but many additional studies are required to completely understand the mechanisms involve.

### A Brief Introduction to the Nigrostriatal Habit Circuitry

The majority of aforementioned DA studies measured its release in the primary terminal field of the mesocorticolimbic pathway (Figure 2C), the NAc. The NAc is typically thought of as a Pavlovian-motor interface that guides model-based goal-directed actions (Yin and Knowlton, 2006; Bornstein and Daw, 2011)—including reward seeking, conditioned active avoidance, and periodically reinforced behavior. In contrast, habits are thought to mediated by interactions between the dorsal striatum and the cortex, or cortico-striatal loops (Figure 2C). The dorsal striatum is often divided into the dorsomedial (caudate in primates) and the dorsolateral (putamen in primates) striatum (Yin and Knowlton, 2006). The dorsomedial striatum is thought to guide model-based, goal-directed actions using environmental rules that dictate the contingencies of reinforcement. By contrast, the dorsolateral striatum is thought to play a role in guiding model-free, habitual action using previously learned associations (Yin and Knowlton, 2006; Lee et al., 2014). This form of model-free habitual action is thought to arise from sensorimotor loops that can be modified by eCB signaling and DA release at the level of the dorsal striatum. Whereas the NAc receives DAergic input from mesocorticolimbic pathway originating in the VTA, the dorsal striatum primarily receives DAergic input from the nigrostriatal pathway originating in the substantia nigra pars compacta (Figure 2C).

### eCB Modulation of Cortico-Striatal Input Into the Dorsal Striatum Gates Goal-Directed and Habitual Behavior

Gremel et al. (2016) provided incisive insight into the role eCBs might play in orbitostriatal input into the dorsal striatum. The orbitofrontal cortex (OFC) is thought to contribute to cortico-striatal loops that may gate behavior between being goal-directed and habitual. Using viral technology to selectively knock-out CB1 from OFC neurons, they first demonstrated that OFC neurons projecting to the dorsomedial striatum exhibit greater activity during goal-directed behavior in a VR than in a VI task. Similar to Hilario, they further confirmed that VI training produced more habitual responding than VR training. They then used a retrograde virus and chemogenetics to selectively inhibit OFC projections into the dorsomedial striatum. While control mice reduced responding on both the VR and VI task when in the devalued (i.e., sugar-sated) state, chemogenetic inhibition of OFC input into the dorsomedial striatum did not reduce responding in either the VR or VI task (Figure 6C). To better assess the local contribution of OFC input in the dorsomedial striatum, they repeated their experiment but injected the clozapine-n-oxide used to induce chemogenetic suppression directly into the dorsal striatum rather than into the intraperitoneal space. They found that handling the WT mice during microinfusions abolished habitual responding; specifically, mice only responded on the sucrose-paired lever when in a sucrose-sated, or devalued state. However, when the microinfusion produced chemogenetic inhibition of OFC input into the dorsal striatum, responding on the previously sucrose-paired lever persisted in-spite of sucrose devaluation. Taken together, these data suggest that eCB-modulation of OFC input into the dorsal striatum might gate behavior between dorsomedial-mediated goal-directed behavior and dorsolateral-mediated habitual behavior. Future studies are necessary to clarify the specific roles distinct eCBs play in modulating behavior; the specific eCBs involved, the circuit they are acting in, and the specific cell-type they are acting on are all important variables to consider in future studies.

### 2AG From D1-Expressing MSNs Mediates Exploratory Behavior and Perseverative Responding

To provide cell-type and eCB specific data, Shonesy et al. (2018) investigated the effects of conditionally knocking down the primary synthetic enzyme of 2AG (i.e., DAGL) from striatal MSNs. The majority of MSNs in the dorsal striatum can be segregated into one of two populations. The D1-expressing neurons of the direct pathway are thought to promote action during reinforcement, whereas the D2-expressing neurons of the indirect pathway are thought to inhibit action during reinforcement (Kravitz et al., 2012). Shonesy et al. (2018) found that knocking down DAGL from the D2-expressing neurons of the indirect pathway failed to influence any of the behavioral outcomes they assessed. In contrast, they found that removing 2AG signaling from D1-expressing neurons of the direct pathway produced distinct behavioral effects depending on whether the conditional knock down occurred in the dorsal or ventral striatum. Specifically, they found that removing 2AG from dorsal striatal D1-containing MSNs reduced both social (Figure 6D) and spatial (Figure 6E) exploration of novelty. However, it should be noted that repetitive grooming occurred following removal of 2AG signaling from the ventral rather than the dorsal striatum. The authors also found that removing 2AG signaling from MSNs failed to influence operant behavior maintained under either fixed-ratio or progressive-ratio schedule. These paradoxical behavioral findings notwithstanding, Shonesy et al. (2018) also provided important information regarding the role of 2AG in modulating synaptic plasticity in striatal circuits. Using electrophysiology, they found that removing 2AG signaling from D1-MSNs reduced feedback inhibition at both glutamatergic and GABAergic MSN synapses and increased basal glutamatergic release onto D1-MSNs. Specifically, they found that KO of DAGL from D1-MSNs significantly increased the frequency of their excitatory post-synaptic currents, suggesting an impairment of eCB mediated feedback inhibition on glutamate release (i.e., DSE). They also found that the KO of DAGL from D1-MSNs impaired DSI at these cells arising from GABAergic synapses, although GABAergic transmission was determined to be unchanged. Overall, these data suggest DAGL-KO from D1-MSNs excite dMSNs due to a loss of DSE. When this breakdown in feedback regulation occurs in the dorsal striatum, exploration of social and spatial novelty are impaired; when this breakdown occurs in the ventral striatum, perseverative grooming behavior is observed.

### Surprising Findings and Important Considerations Regarding the Overlap Between Measures of Appetitive Goal-Seeking and Habitual Responding

While the aforementioned studies offer compelling evidence that increases in 2AG within the dorsal striatum act on CB1s to modulate habitual behavior, a recent study by Gianessi et al. (2021), suggest there is considerable nuance to this story that still needs to be considered. These investigators trained mice to respond for sucrose-sweetened grain pellets under a VI schedule and then tested for habitual responding using contingency degradation. As opposed to sating the mice with *ad libitum* sugar, the reinforcement contingency was degraded by allowing the animal to respond as if in the VI-task but lever presses resulted in no programmed consequence. Rather, reinforcers were delivered at equal intervals, matching the total number of reinforcers earned the previous day. The FAAH inhibitor URB597 was administered to test the effects of elevated AEA levels on habitual responding; the MAGL inhibitor JZL184 was administered to test the effects of elevated 2AG levels on habitual responding. Contrary to their predictions, they found that both drugs reduced responding during the test day following contingency degradation (Figure 6F). These findings paradoxically suggest that elevating neither anandamide nor 2AG strengthens habitual responding. Furthermore, they demonstrated that the effects of the CB1 antagonist/inverse agonist AM251 on habitual responding varied depending on the vehicle used and the relative time of drug pre-treatment. The authors first note that solubility of prepared cannabinoid solutions varies greatly across labs because these lipophilic compounds are not easily dissolved in water. They demonstrate that dissolving AM251 in a mixture of DMSO and TWEEN produced dose-dependent reductions in operant responding, but dissolving AM251 in DMSO alone did not. Thus, it is important to note differences in vehicle and drug preparation may drastically impact bioavailability when comparing cannabinoid studies. For example, while the Gianessi et al., 2021 study reported that 1 mg/kg AM251 reduced operant responding, the Hilario study reported that neither 3 nor 6 mg/kg AM251 did. Perhaps more importantly, Gianessi et al., 2019., also found that the timing of habitual testing relative to drug-treatment is important to consider during experimental design. When they assessed for habitual behavior immediately after a series of AM251 treatments, they observed a significant increase in responding. However, when they assessed for habitual behavior after allowing for AM251 to clear the system, responding was found to be decreased. From this observation, and their finding that AM251 reduced responding for sugar pellets, the authors concluded that mice increased responding after the series of AM251 treatments because they had not been reaching satiety across the VI training sessions and were therefore showing an increase in goal-directed appetitive behavior rather than habitual responding during the first contingency degradation session. It is also worth noting that this group also reported that when administered alone, JZL184 does not alter the expression of food habits (Gianessi et al., 2019) or alcohol habits (Gianessi et al., 2020). However, in the latter study Gianessi et al. (2020) did find that JZL184 increased motivation for food as assessed using a progressive ratio schedule. Thus, while compelling evidence suggests that 2AG may be important in gating goal-directed to habitual action, many more studies are required to reconcile the nodes of the neural circuitry involved, the role of specific receptors and cell-types being acted upon within each node, and the contributions of distinct eCBs. Furthermore, the potential confound of CB1-mediated changes in appetitive behavior on habitual testing underscores the importance of concurrently considering the literature on eCB-modulation of appetitive behaviors, habitual responding, and attentional processes.

### Transition From Reinforcement to Attentional Processes

The manifestation of motivationally switching from a primary reinforcer to an alternative outcome and habit-formation likely involve the additional recruitment of attentional processes. And, when considering the neural substrates involved in motivational switching, it became readily apparent that this circuitry often overlaps with the neural substrates of attention (e.g., OFC-dorsal striatum) (O’Hare et al., 2018). Furthermore, mesocorticolimbic DA signaling is believed to modulate value-driven goal-directed action, habit formation, and attentional processes. Thus, we next turn the focus of our review to the seemingly intertwined literature on cannabinoid and DAergic modulation of attentional processes.

## Cannabinoids and DA Both Modulate Attentional Processes as Well

### Introduction to the Study of Attention and Attentional Processes

The concept of attention has long historical roots in psychology and bears several definitions. While modern terminology surrounding attention may refer to disparate concepts such as arousal, vigilance, and distractibility, it may be broadly defined as selective activation of neural representations during information processing. Through this definition, attention may be best illustrated in relation to the highly related process of working memory. Whereas attention uploads information ‘on-line’ at any discrete timepoint, working memory stores and utilizes these activated representations during recall across small spans of time (Baddeley, 1986; Cowan, 1993; McElree, 2001; Oberauer, 2019). While attention has different aspects or components associated with it, including its most fundamental sensory-based component involuntarily elicited in response to salient environmental stimuli, the behavioral paradigms referenced below generally focus on attentional control. An executive function, attentional control incorporates top-down regulation of bottom-up sensory driven attentional processes to subserve appropriate attendance toward behaviorally relevant stimuli (Posner and Petersen, 1990; Cohen et al., 1993; Hopfinger et al., 2000; Fan et al., 2002). Proper allocation of attention within complex, changing environments is an evolutionarily conserved trait crucial for effective information processing (Matzel and Kolata, 2010; Chun et al., 2011), allowing an animal’s behavior to be adaptively modified by external contingencies in order to successfully engage in signal detection and goal-directed decision making (Broadbent and Gregory, 1963; Endler, 1992; Verghese, 2001; Smith and Ratcliff, 2009; Asplund et al., 2010; Voloh et al., 2015). So, dysfunctions in attention weaken an individual’s ability to allocate cognitive resources effectively to the task at hand. Therefore, deficits in attentional control are potential barriers to adaptive behavior and overall survivability of the organism, with pathologies affecting this executive function leading to maladaptive traits that negatively impact quality of life (Baddeley et al., 2001; Rueda et al., 2004; Williams-Gray et al., 2008; Burgess et al., 2010; Fajkowska and Derryberry, 2010; Schoorl et al., 2014; Stefanopoulou et al., 2014; Heeren and McNally, 2016).

### Cortical Regulation of Attentional Control

Although the neuroanatomical loci of attention are many and work as an integrated network of multiple brain regions, attentional control is largely mediated by cortical regions. Spatial and visual attentional control, for instance, have been evidenced to be strongly regulated by frontoparietal regions that filter sensory information in a top-down fashion, with injury to these areas resulting in spatial neglect despite intact bottom-up, sensory-driven networks (Jeannerod, 1987; Karnath et al., 2001; Mort et al., 2003; Corbetta et al., 2005; Fiebelkorn et al., 2018). In terms of attentional command and action selection, the PFC and OFC have been shown to mediate selective attentional control during cognitive tasks, with the PFC regulating attentional focus during interference (Milham et al., 2001), redirection of attention based on task demands (Rossi et al., 2007), and attentional shifting across perceptual features (Owen et al., 1991; Birrell and Brown, 2000; Liston et al., 2009), while the OFC primarily serves redirecting attention during reinforcement switching within reversal learning (Hampshire and Owen, 2006). As the PFC is fundamental to cognitive control in general and regulates working memory, decision making, and other processes crucial to goal-directed behavior (Fuster, 2015), its involvement in attentional processes is perhaps self-evident. The OFC, on the other hand, has a more indirect relationship to attention as it is more associated with value encoding and behavioral inhibition (Teitelbaum, 1964; Gallagher et al., 1999; Izquierdo et al., 2004; Kim and Ragozzino, 2005; Jonker et al., 2015). Nonetheless, attention-based modulation of value encoding in the OFC has been recently supported, leaving an interesting role for the OFC in value-based decision making that may be under the control of attentional focus (Xie et al., 2018).

### DA and eCB Regulation of Cortical Function

The multifaceted cortical functions of cognitive control are tightly regulated by both intra- and intercortical activity states mediated greatly by pyramidal cells, the principal neurons of the cortex. Far from being self-contained, pyramidal cell activity is impinged by numerous signaling molecules, including DA, which is projected in the cortex by rich innervations arising from the VTA (Lewis et al., 1986). DA regulates pyramidal cell function through numerous ways to primarily modulate glutamatergic and GABAergic signaling in the cortex (Law-Tho et al., 1994; Zheng et al., 1999; Gao et al., 2001; Seamans et al., 2001a; Flores-Hernandez et al., 2002; Gao and Goldman-Rakic, 2003; Wang et al., 2003; Beazely et al., 2006; Liu et al., 2006; Onn et al., 2006; Li et al., 2009; Hu et al., 2010; Tritsch and Sabatini, 2012). Overall DA has a dampening effect on excitatory transmission in the PFC through a presynaptic mechanism, reducing the probability of glutamate release (Gao et al., 2001). DA also modulates inhibitory signaling in the PFC, biphasically altering inhibition of pyramidal cells via G_*i*_-coupled D2 DA receptor activation on presynaptic GABA cells and a complex interplay between signaling of postsynaptic pyramidal cell DA receptors D1 (G_*s*_-coupled), D2, and D4 (Gi-coupled) (Seamans et al., 2001b; Wang et al., 2002; Trantham-Davidson et al., 2004). Pyramidal cell activity is modulated by DA in more direct ways too; postsynaptic mechanisms of intrinsic excitability have been shown to be adjusted in rats by VTA DA projections that modify spike frequency adaption and afterhyperpolarization potentials in the PFC (Buchta et al., 2017).

Cortical function is also mediated by eCB signaling. In the PFC, CB1 expression has been found to be preferential to GABAergic presynaptic terminals adjacent to glutamatergic ones, both synapsing onto dendrites of mGluR5-containing pyramidal cells. This places CB1 in a position to integrate and balance excitatory and inhibitory signaling during activity-dependent eCB mobilization (Fitzgerald et al., 2019). This mGluR5-mediated integration of PFC pyramidal signaling may take place post-synaptically to directly increase pyramidal cell excitability and synaptic drive, or pre-synaptically as this ligand gated Gq protein-coupled receptor is capable of stimulating eCB production to induce DSI-mediated disinhibition of pyramidal cells via CB1 signaling (Kiritoshi et al., 2013). eCB signaling may also simultaneously modulate glutamate and DA in the PFC as systemic administration of the CB1 agonist WIN has been shown to increase transmission of both within this region (Polissidis et al., 2013). Furthermore, intra-PFC WIN administration induces bi-phasic functional effects in VTA DA cell activity, with low doses increasing and high doses decreasing spontaneous DA cell firing (Draycott et al., 2014). Although less characterized, eCB signaling within the OFC influences pyramidal function too. Similar to the PFC, postsynaptic mGluR5 activation has been shown to increase local eCB release and enhance CB1 signaling within GABAergic presynaptic terminals of the OFC (Lau et al., 2020). Interestingly, in the lateral aspect of the OFC, impaired astrocytic glutamate transport has been found to result in aberrant eCB tone and subsequent LTD of inhibition onto pyramidal cells, presumably via increased mGluR5 activation from excess extrasynaptic glutamate. Whether this eCB-mediated astrocytic regulation of mGluR5 activation is shared by PFC synapses remains to be investigated.

### DA and eCB Regulation of Attentional Processes

The influence of cortical DA on cognition, including attentional processes, is a well-researched subject that has been intensely studied by neurobiologists and computational neuroscientists alike. Broadly, DA in the PFC facilitates integration of complex signals between sensorimotor networks by synchronizing different brain networks in response to both external signals and internal representations (Ott and Nieder, 2019). This is enabled by stabilizing neural representations in the cortex through gating sensory signals at the level of the PFC and gain changes of different pyramidal cell subpopulations, which support action selection and goal-directed behavior in stimuli-rich environments (Foote et al., 1975; Durstewitz et al., 2000; Mehta et al., 2000; Assad, 2003; Yantis and Serences, 2003; Maunsell and Treue, 2006; Scolari and Serences, 2009; Dang et al., 2012; Byers and Serences, 2014; Shafiei et al., 2019). As a neuromodulator, DA’s influence via the signaling dynamics referenced above are tightly regulated at both the synaptic and systems level and are subject to the classic Yerkes-Dodson (inverted U-shaped curve) dose-response relationship, with hyper- or hypoDAergic levels resulting in cognitive dysfunction (Yerkes and Dodson, 1908; Vijayraghavan et al., 2007). This DA-sensitive nature of attentional control has been demonstrated by both human and rodent studies showing measures of inattentiveness correlated with low levels of DA release may be repaired by increasing DA transmission by means of neural stimulation or pharmacological manipulation (Turner et al., 2017; Fukai et al., 2019). In contrast, administering the D2 antagonist haloperidol to healthy human subjects increases involuntary directing of attention toward task-irrelevant events (Kähkönen et al., 2002). eCB signaling within the cortex must also walk a fine line to sustain attentional control and while local cortical CB1 dynamics are less studied than those within cortico-accumbens projections within this context, their effect on cognition is duly noted. In the PFC, viral-induced overexpression of CB1 results in impaired cognitive flexibility in the form of decreased reversal learning in rats (Klugmann et al., 2011). Within the OFC, the medial but not the lateral aspect has been found to display low levels of CB1 gene expression in rats with high impulsivity (Ucha et al., 2019). And, goal-directed behavior in mice has been shown to be regulated by a CB1-dependent mechanism in OFC projections to the dorsal striatum, with genetic knock out of CB1 here preventing habit formation of instrumental responding (Gremel et al., 2016).

### Common Methods to Investigate the Components of Attention

While there are many components of attentional control, this review will focus on sustained attention, response control (impulsivity), attentional set-shifting and reversal learning as indices of attentional control as well as their respective deficits. Of the many factors that may influence attentional control, reversal learning – instrumental responding to swapped outcome contingencies between manipulanda – and impulsivity are both affective state-sensitive, pathology-related variables readily examined in the operant setting as adjuncts to more direct measures of attention itself (Puumala and Sirviö, 1998; Kenemans et al., 2005; Izquierdo and Jentsch, 2012; Linley et al., 2016; Paret and Bublatzky, 2020). While these two measures remain technically distinct from those of attention *per se*, they index prioritization of attentional demand to reward-associated stimuli (Mackintosh and Little, 1969; Oemisch et al., 2017). Accordingly, their relationship to attention and its operant tests are discussed alongside attention itself. Because performance inconsistencies are more informative than absolute performance, and because anatomical and neurochemical specificity is more readily correlated to specific measures, focus will be given to impairments and enhancements of these measures under different pharmacological conditions. Limitations are inherent in each reported finding as no pure test of attention is currently accepted, although evidence supporting correlations between certain pathologies and specific attentional dysfunctions will be highlighted. As attention is both inherently sensitive and limited, unique internal (e.g., neurofunctional) and external (e.g., experiential) factors may affect its processing to either enhance or constrain different attentional components. Furthermore, individual differences in attentional control may result in differing perceptions and behavioral outputs across samples under identical environmental conditions (Dukas and Kamil, 2000; Derryberry and Reed, 2002; Mathews et al., 2004; Ólafsson et al., 2011; Sali et al., 2015; Yuan et al., 2019). Such factors will be considered here, focusing on how key mesocorticolimbic regions regulate commonly investigated attentional control processes while also relating changes in functional activity to pathology. The review will then culminate with DA/eCB interactions evidenced to modulate these processes with special consideration toward gaps in the literature. To best frame the aforementioned components of attention, the behavioral tests most popularly used for their measurement will be introduced below, with the 5-Choice Serial Reaction Time Test (5-CSRTT) used to assess sustained attention and impulsivity and the Attentional Set Shifting Test (ASST) used to measure shifting attentional set and reversal learning.

### Operant Methods to Assess for Changes in Attentional Processes

#### The 5-Choice Serial Reaction Time Test (5-CSRTT)

**FIGURE 7:**
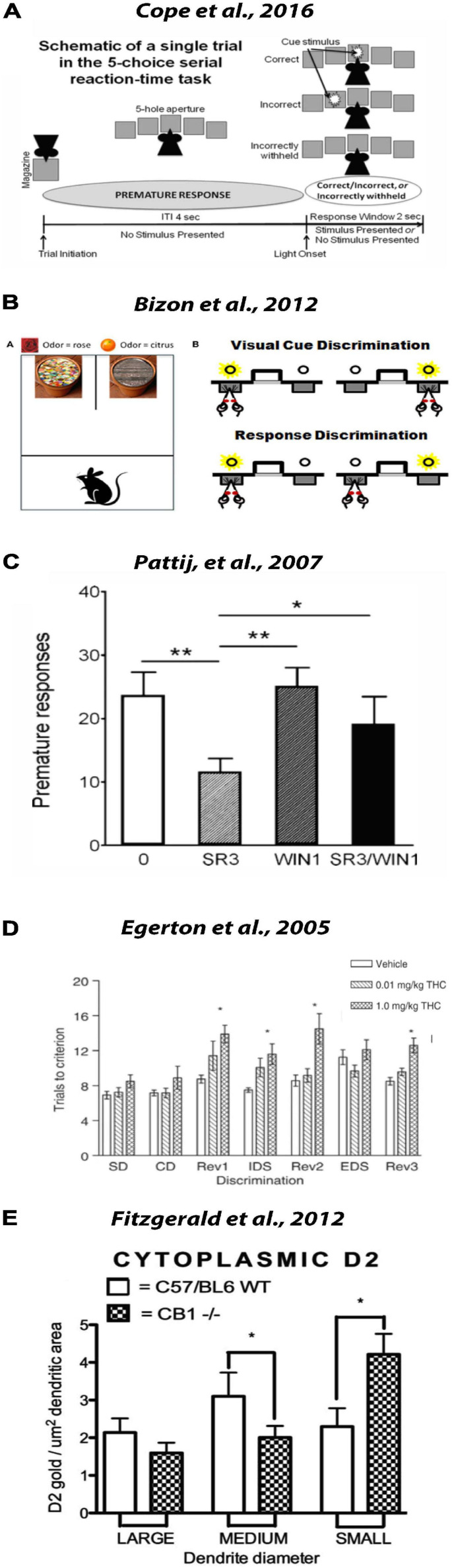
Cannabinoids modulate attentional processes. **(A)** Schematic of a single trial in the 5-choice serial reaction time task (5-CSRTT). Animals are trained to perform a nose poke when one of five cue lights is presented in any of the five nose-poke apertures following a fixed inter-trial interval (ITI), usually 4–6 s in length. Premature responses are tallied when made during this ITI. Responses made in one of the four nose pokes not illuminated is counted as an error of sustained attention. Reproduced from Cope et al. (2016). **(B)** Task schematics of two common set-shifting assessments for rodents. Panel **(A)** shows a schematic of the apparatus and examples of the stimuli used in the “dig” set-shifting task. Each pot has a unique odor (i.e., rose on left and citrus on right) and is filled with a unique digging medium (sequins on left, gravel on right). Only one stimulus feature is relevant to the location of a buried food reward in each phase of testing. Panel **(B)** shows a schematic of set-shift procedures performed in an operant version of the task. Rats are first trained to choose between two extended levers based on a light cue that is associated with one of the levers. After reaching criterion performance on that discrimination, there is an unsignaled change in rule and now the rat must ignore the light and choose levers based on their spatial location. Reproduced from Bizon et al. (2012). **(C)** Effect of CB1 antagonist/reverse agonist on impulsivity. Coadministration of WIN55,212-2 at 1.0 mg/kg (WIN1) prevents the effects of 3.0 mg/kg SR14716A (SR3) on inhibitory control in the 5-CSRTT. Reproduced from Pattij et al. (2007a). **(D)** Effect of acute THC administration on reversal learning. At 30 min before the start of the task, rats were administered vehicle, 0.01 mg/kg THC, or 1.0 mg/kg THC and the number of trials to reach criterion performance was recorded for a series of discriminations (SD, simple discrimination; CD, compound discrimination; Rev1,2,3, first, second, and third reversal stages; IDS, intradimensional shift; EDS, extradimensional shift). Animals in the 1 mg/kg THC treatment group exhibited marked deficits in performance at each of the reversal stages but not in the EDS stage. Reproduced from Egerton et al. (2005). **(E)** Altered compartmentalization of D2 immunogold stain in dendrites containing immunoperoxidase labeling for parvalbumin in the PL of the CB1^–/–^ mice. Cluster analysis reveals a significant change in compartmental distribution of D2 immunogold in parvalbumin dendrites of CB1^–/–^ mice. D2 immunogold density was assessed as particles of D2 immunogold/square μm dendritic area. In CB1^–/–^ mice relative to CB1^+/+^ controls, a significant (*p* < 0.05) increase in D2 immunogold was observed in small dendrites, while a decrease in D2 immunogold per μm dendritic area was observed in medium parvalbumin dendrites in CB1^–/–^ mice relative to controls. Reproduced from Fitzgerald et al. (2012). *p < 0.01; **p ≤ 0.001.

The 5-CSRTT for rodents was refurbished from a similar test of attentional processing during discrimination of visual stimuli in humans (Wilkinson, 1963; Carli et al., 1983). The paradigm consists of a food cup positioned in front of a hinged window, which once pushed open by the rodent initiates the behavioral session and delivery of the first food-based reward, usually a food pellet or measured amount of liquid sucrose. Additional manipulanda consists of five nose poke ports, each with their own cue lights positioned behind them as well as photobeams to detect individual nose pokes. After initial reward delivery following the opening of the food cup window, each successive delivery of reward is contingent upon a successful, exclusive nose poke through the port in which a cue light is randomly illuminated per trial. Responses for any port not signaled with an illuminated cue light may either terminate the trial without reward delivery or be tallied as non-rewarding errors within a lengthened response period (depending on the behavioral script ran at the time), after which the next trial begins. As the cue light is only briefly illuminated and responding via nose pokes is only allowed during a confined time period, a temporal domain is imposed onto the spatial domain defined by the five different manipulanda separated by the apparatus. This dual-domain aspect of the paradigm demands attention be afforded to both domains simultaneously but also allows experimenters to dissociate each as they see fit, for instance by expanding the spatial separation of cues by exclusively illuminating peripheral ports or modifying time periods of cue illumination and/or delay periods. The dual-domain component of the task also allows multiple aspects of attention to be measured within a single experimental session. For instance, errors counted across nose-poke responses within unilluminated ports, considered inaccurate responses, are interpreted as lapses in sustained attention. Additional demands of ‘attentional load’ placed on the animal may be measured by modifying the temporal domain to increase uncertainty and/or duration of cue illumination. Another type of error may also be measured by tallying responses made during a brief inter-trial interval period programed before cue illumination at the onset of each trial (Figure 7A). These premature responses are interpreted as lapses in inhibitory control, or impulsivity. Other types of errors may also be measured by additional modifications programmed into the paradigm, though this review will focus on those of inaccurate and premature responding as a bulk of literature supports both DA and eCBs mediate these aspects of the task as commonly used with rodents.

#### The Attentional Set Shifting Test (ASST)

Like the 5-CSRTT, the ASST for rodents was adapted from behavioral assessments originally designed for human subjects. The most commonly cited comparison is with the Wisconsin Card Sorting Test (WCST), though a more direct comparison may be made with the Cambridge Neuropsychological Automated Testing Battery (CANTAB). Both are used to investigate ‘behavioral flexibility’ in healthy and abnormal neurological states by measuring the ability to shift attention from one reward-predictive perceptual feature to another following an unexpected switch (Berg, 1948; Grant and Berg, 1948; Weinberger et al., 1986; Sahakian and Owen, 1992; Paolo et al., 1995; West, 1996; Nieuwenstein et al., 2001; Barceló and Knight, 2002; Ridderinkhof et al., 2002; Romine et al., 2004; Nagahama et al., 2005). Specifically, the CANTAB design involves two-choice discriminations between either simple or complex exemplars to assess attentional bias toward a feature of perceptual stimuli, or dimension. One exemplar initially predicts reward faithfully at onset of the task and constitutes a single dimension (e.g., shape), while the other non-predictive exemplar is a presentation of a separate stimulus within the same dimension, in this case a separate shape. Once subjects learn this simple discrimination, a complex discrimination must be made after introduction of a second dimension (e.g., line segments) overlaying the first dimension in each exemplar that remains reward-predictive (shape). As both exemplars now consist of separate stimuli constituting two different dimensions (e.g., two different shapes with two superimposed line segments), the reward-predictive stimuli may be ‘shifted’ within the same dimension or to the other dimension. Changing stimuli while retaining reward-predictability to the initial dimension (shape) is labeled an ‘intradimensional shift’ (ID shift), while switching the reward-predictive dimension (shape→line segments) is labeled an ‘extradimensional shift’ (ED shift). Adding another level of analysis, each of these test components is followed by a reversal learning test, in which the reward-predictive stimuli of the two exemplars is reversed while the relevant dimension stays the same.

The ASST adapted for rodents has been designed as both a reward-digging task, in which exemplars comprise different combinations of digging materials and odors as dimensions (Figure 7B, panel A), and an instrumental operant task, in which reward delivery associations may be switched between two different levers and their respective cue light illumination settings (on/off) (Figure 7B, panel B). In these tasks, a bias toward one of the two dimensions is considered formation of an attentional set, expressed as relatively quicker and more accurate responding during intradimensional shifts than either extradimensional shifts or the initial simple discrimination test (Birrell and Brown, 2000). Attentional sets are therefore interpreted as information stores maintaining the reward-associative value of a perceptual feature that leads to relative ignorance toward other features (Folk et al., 1992).

In addition to assessing attentional sets, the separate reversal learning tests allow dissociation and detection of deficits relating to this ability alone. While similar to attentional set-shifting, reversal learning is considered a less complex but important process that relies on inhibition of previously rewarding actions (Jones and Mishkin, 1972). Because shifting attentional set requires a higher demand of attentional orientation and an aspect of learned irrelevance – accurately responding to rearrangement of complex, multidimensional stimulus pairings not correlated with reward – it is widely considered to be more cognitively challenging than reversal learning (Dias et al., 1996; Bissonette et al., 2008; Nilsson et al., 2015). Owing to its regulation of complex cognitive functions, it is logical that the PFC has been found to be critical to attentional set-shifting, with the non-human primate lateral PFC and homologous rodent medial PFC specifically evidenced to mediate this process (Dias et al., 1996; Birrell and Brown, 2000). Conversely, reversal learning is specifically impaired following damage to the OFC of both monkeys and rodents, perhaps owing to this region’s encoding of reward value during decision-making processes that may influence response inhibition toward previously rewarding actions (Dias et al., 1997; McAlonan and Brown, 2003).

### DA and eCB Signaling Effects on 5-CSRTT Performance

While both mesocorticolimbic DA and eCB signaling have been shown to affect 5-CSRTT performance, their influence on sustained attention and impulsivity may be separable. DA has been shown to play a crucial role in controlling inhibitory responding, with converging data indicating elevating synaptic DA increases impulsivity by activation of both D1 and D2 receptors (van Gaalen et al., 2006; Baarendse and Vanderschuren, 2012; Xue et al., 2018). In rodents, impulsivity has been linked to DA signaling specifically within the medial PFC and NAc core and shell (Cole and Robbins, 1987; Miller and Cohen, 2001; Chudasama and Robbins, 2004; Dalley et al., 2004; Economidou et al., 2012). eCB tone may also be primed to modulate impulsivity as the synthetic CB1 antagonist rimonabant dose-dependently decrease it in rats, an effect occluded by co-administered WIN 55 (Pattij et al., 2007a) (Figure 7C). CB1 activity likely mediates its influence on impulsivity through regulating DA signaling as the CB1 antagonist rimonabant dose-dependently attenuates the impulsivity-inducing effects of the psychostimulants d-amphetamine and cocaine (Wiskerke et al., 2011; Hernandez et al., 2014). Additionally, THC and WIN administered twice daily across 2 weeks results in reduced DA turnover exclusively in the PFC and not in the dorsal nor ventral striatum of rats, with effects lasting up to at least 14 days post-abstinence (Verrico et al., 2003). This study is interesting in light of evidence supporting that response inhibition is regulated by cortical substrates and that protracted abstinence from chronic THC administration selectively impairs response inhibition in rats (Eagle and Baunez, 2010; Irimia et al., 2015). Collectively, these data suggest that following chronic cannabinoid exposure, long-term adaptations of DA function within the PFC increase likelihood of impulsivity.

In contrast to CB1-dependent effects on impulsivity, cannabinergic effects on more direct measures of attention in the 5-CSRTT are relatively null. Accordingly, modest impairments in sustained attention have been found to be reversed after 2 weeks abstinence following chronic THC administration in rats, with more pronounced increases in impulsivity persisting after 5 weeks of abstinence (Irimia et al., 2015). The distinction between impulsivity- and sustained attention-related effects of DA signaling have been scrutinized more than those by eCB signaling though results are conflicting. DA-dependent effects on 5-CSRTT performance differ based on pharmacological modality of altered activity (chemogenetics vs. psychostimulants), individual differences in baseline task performance between subjects and region-specificity of manipulations made within the mesocorticolimbic system. These variables notwithstanding, evidence reveals DA signaling regulates sustained attention albeit to a lesser degree than impulsivity. In general, sustained attention is enhanced following local D1 receptor agonism in the medial PFC and NAc as well as after increased neuronal activation of the VTA via selective pharmacological manipulation of modified G_*i*_-coupled muscarinic GPCRs (i.e., chemogenetics) (Granon et al., 2000; van Gaalen et al., 2006; Pattij et al., 2007b; Baarendse and Vanderschuren, 2012; Boekhoudt et al., 2017; Xue et al., 2018; Fitzpatrick et al., 2019). Yet, multiple pharmacological and biological variables must be accounted for when manipulating DA function for behavioral output in general, though perhaps more so with cognitive tasks susceptible to numerous factors. Further dissecting any dissociable effects of chemogenetics from other types of DAergic manipulations, as well differentiating region-specific effects, may prove useful to probe DA’s attentional functions within the 5-CSRTT.

### DA and eCB Signaling Effects on ASST Performance

The effects of both mesocorticolimbic DA and eCB signaling on ASST performance diverge by the task’s separate components, with DA impacting both shifting attentional set and reversal learning and cannabinergic effects restricted to reversal learning. Converging lines of evidence suggest that D1 signaling within the medial PFC in rodents and the homologous DLPFC in primates is central to attentional set formation and shifting in the ASST (Dias et al., 1997; Ragozzino et al., 1999; Birrell and Brown, 2000; Stuss et al., 2000; Stefani et al., 2003; Tunbridge et al., 2004; Fletcher et al., 2005; Floresco et al., 2008; Nagano-Saito et al., 2008; Parsegian et al., 2011) while D2 and DAT signaling within the OFC and striatum support reversal learning, respectively (Cools et al., 2009; Izquierdo et al., 2010; Cheng and Li, 2013). Indeed, cognitive flexibility training has been shown to enhance measures of prelimbic DA and therapeutic cognitive benefits in rats (Chaby et al., 2019), while ADHD and schizophrenia patients, both strongly associated with dysregulated cortical DA function, display attentional dysfunctions particularly related to shifting attentional set similar to patients with frontal lobe damage (Pantelis et al., 1999; Luna-Rodriguez et al., 2018). In terms of OFC DA, low but not high doses of methylphenidate remediate the impairment of both attentional-set formation and reversal learning in the spontaneously hypertensive rat (SHR) model of ADHD (Cao et al., 2012), though this effect on reversal learning specifically is occluded by intra-OFC injections of the D2 antagonist haloperidol (Cheng and Li, 2013).

Insight into the cannabinergic effects on ASST, on the other hand, are far outnumbered by those of DA, though these limited studies suggest eCBs may be more important for reversal learning than shifting attentional set. Egerton et al. (2005) first demonstrated that acute THC in rats impairs reversal learning while sparing extradimensional set-shifting ability in the ASST (Figure 7D). This selective effect on reversal learning by THC has been corroborated in non-human primates in the CANTAB test (WrightJr., Vandewater et al., 2013). Reversal learning has also been shown to be impaired in rats by THC in an olfactory go/no-go discrimination task and by adolescent WIN exposure in the ASST (Sokolic et al., 2011; Gomes et al., 2015). Surprisingly, cannabinoid-induced deficits to ASST may violate notions of a PFC/OFC task-specific dichotomy as overexpression of CB1 specific to the medial PFC has been shown to selectively impair reversal learning in rats, a cognitive component of the ASST typically associated with OFC function (Klugmann et al., 2011). Also surprising is a recent finding that intra-PFC injection of cannabidiol (CBD), but not similarly administered THC, impairs shifting of attentional set in rats (Szkudlarek et al., 2019). While its pharmacodynamic profile is complicated, it is worth noting that CBD functions as a negative allosteric modulator at CB1 (Laprairie et al., 2015).

Finally, several studies using rats prenatally treated with methylazoxymethanol acetate (MAM) as a developmental model of schizophrenia suggest persistent psychotomimetic effects related to mesocortical neuroadaptations may result from aberrant eCB signaling during adolescence (Renard et al., 2017). Both MAM and pubertal WIN have been shown to impair reversal learning in the ASST, as well as enhance mobility effects of d-amphetamine administration and an increased number of spontaneously active VTA DA neurons (Gomes et al., 2015). Somewhat remarkably, cannabinoid effects on MAM treatment are suggested to be transgenerational as adolescent WIN exposure also increases VTA DA population activity, decreased burst firing and sensitization to d-amphetamine locomotor responses in Figure 2 generation MAM-treated rats (Aguilar et al., 2018).

These results bring into question both the locus of reversal learning and the neural mechanisms underlining its impairment by cannabinoids. Also important are considerations of dissimilarities between DA manipulations and ASST models, since like most laboratory-controlled behaviors, performance variations may be attributed to different protocols. This is underlined by cannabinoid administration having been suggested to impact visual discrimination in general, which may broadly affect performance in operant chamber-based ASST paradigms (Arguello and Jentsch, 2004; Hill et al., 2006). Future research of reversal learning following cannabinoid exposure and manipulation should take such details into account.

### Comparable Studies Targeting Separate Signaling Systems May Benefit Analysis of DA/eCB Interaction and Attentional Dysfunction

To assist in clarifying DA/eCB interactions and their effects on attention, a few effective approaches may be noted. Behavioral effects of DA/eCB interactions have been demonstrated combining targeted pharmacological manipulations with measures of negative affect, showing anxiolytic effects of CB1 activation in the amygdala is D1 and D2 dependent (Zarrindast et al., 2011). Additional work with conditional knock-out mice with CB1 expression constitutively removed from D1-expressing neurons revealed CB1/D1 interactions modify negative affect as well (Terzian et al., 2011). Another comparative pharmacology study reported acute cannabis decreases while cocaine increases reversal learning performance in human subjects (Spronk et al., 2016). Interestingly, recent data combining chemical lesions of the medial forebrain bundle and single-unit electrophysiological recordings suggests the hypoDAergic states associated with many neuropsychiatric disorders affecting attention may themselves cause impairments in CB1 functional modulation of both sensorimotor and executive networks (Antonazzo et al., 2020). This novel conception expands the operative role for DA as not only a modulator of glutamate and GABA transmission, but also as a newfound gatekeeper of CB1’s own robust modulation of transmission in substrates important for attentional and behavioral control. Future studies in the causality of CB1 functional modification within DA-affected pathologies and related changes to attentional processes are certainly warranted.

Finally, DA/eCB interactions may also be investigated at the level of GABAergic interneurons. Many of the previously described alterations to mesocorticolimbic functional dynamics may involve FSI that are either directly targeted by eCBs or are ultimately subject to eCB-dependent adaptations, with attentional processing being impacted by both. In the PFC, parvalbumin expressing FSIs critically gate pyramidal activity and are sensitive to DA signaling. Furthermore, levels of dendritic D2 expression in PFC parvalbumin cells have been demonstrated to be regulated by CB1 signaling (Fitzgerald et al., 2012) (Figure 7E). CB1-expressing FSIs in the striatum are also associated with cognitive functions, strongly associated with impulsive behavior and most recently suggested to gate attention toward reward-predicting visual features (Caprioli et al., 2014; Wright et al., 2017; Pisansky et al., 2019; Boroujeni et al., 2020). As eCB-sensitive FSIs in both cortical and striatal regions hold strong potential as a nexus for DA/eCB overlap, studies investigating their role in cannabinoid-modulated attentional processes may clarify much detail lacking in the field.

## Conclusion

### Summary of Conclusion

In this review, we describe recent scientific studies suggesting cannabinoids modulate transient DA release events in a manner that may influence motivational and attentional processes alike. While we’ve acknowledged that DA/eCB interactions still need to be better investigated across multiple overlapping neural circuits, we would like to close by further considering the intertwined relationship between DA transients, motivation and attention. And finally, we offer some speculation into the clinical implications these findings may offer the treatment of neurobehavioral symptoms in psychiatric medicine.

### Complex Interactions Complicate the Relationship Between DA and the Neural Circuitry of Motivation and Attention

Transient DA release events, the neural substrates of motivation, and the neural substrates of attention interact within a tangled thicket of intertwined circuits—the nodes of which likely influence each other and can be differentially modulated by eCBs at multiple levels. First, it is important to recognize that activation of either subcortical nodes of pre-attentive visual processing (e.g., the superior colliculus) or cortical nodes of attentional visual processing (e.g., V1) are sufficient to evoke transient DA release events in the striatum of the basal ganglia (Redgrave et al., 2008, 2016; Takakuwa et al., 2017, 2018). Thus, it becomes difficult to definitively know whether a striatal DA transient truly reflects the value of a desired outcome within a motivational context (as was the general assumption of this review), is the result of an animal responding to a pre-attentive visual stimulus or is the result of an animal giving a visual stimulus attentional consideration. Furthermore, it is becoming abundantly clear that DA transient release events are accompanied by the co-release of additional neurotransmitters (e.g., GABA, glutamate) from the same DA neuron, which may profoundly impact the post-synaptic effects of DA (Tritsch et al., 2016; Morales and Margolis, 2017). In addition, a wide array of discrete neural circuits converge on midbrain DA neurons and their striatal-terminals within the basal ganglia (Morales and Margolis, 2017). It is likely that these discrete circuits can be differentially modulated by eCBs to influence neural input onto DA neurons, the generation of action potentials within DA neurons, and/or the concentrations of neurotransmitter released from the terminals of DA neurons. Following this multifaceted level of modulation, the transient DA release events are then integrated with other neural signals encoding various functions of motivation and attention within nuclei of the basal ganglia to ultimately influence the generation of behavioral action (Den Ouden et al., 2012). Difficulty in dissociating such neural representations has been considered before and ascribed to confounding neural signals of reward expectancy and attentional allocation (Maunsell, 2004). Indeed, DA value signals and motivational states are commonly recognized variables that capably modulate shifts in attention (Engelmann and Pessoa, 2007; Mohanty et al., 2008; Sali et al., 2014; Bourgeois et al., 2016; Anderson, 2019). And, at the level of behavioral output, common measures of both motivation and attention are highly DA-sensitive. This makes dissociating DA’s contribution to their different components somewhat difficult as most experimental assessments of attention are dependent on the subject’s motivation. Take, for example, individual differences in DA function and their effect on cognitive measures demanding attention in a clinical setting. Healthy individuals with relatively lower DA synthesis capacity have been found to exert relatively low cognitive effort, while increasing their DA levels with methylphenidate and the D2 antagonist sulpiride has been reported to enhance their reward perception and motivation for cognitive engagement (Westbrook et al., 2020). This observation of a low DA-low effort relationship may be applied to preclinical settings and aligns with a study in mice reporting that chemogenetic inhibition of VTA DA neurons decreased motivated responding in a 5-CSRTT but not measures of attentional processing *per se*—suggesting an apparent dissociation (Fitzpatrick et al., 2019). However, it has also been reported that increasing DA levels through chemogenetic excitation of the VTA, using selective activation of modified G_*q*_-coupled muscarinic GPCRs, impairs sustained attention in the 5-CSRTT (Boekhoudt et al., 2017). While separate types of neural DA manipulations were utilized between these studies (cf. inhibitory G_*i*_ vs. excitatory G_*q*_ DREADDs), the seeming contradiction may indicate the difficulty in separating the components of motivation and attention by performing DA manipulations in operant tasks.

### Clinical Implications and Considerations

The interrelated nature of motivation and attention may be an asset to research as much as a liability, and cannabinoid modulation of either may underline therapeutic targets for both constructs. In the clinical context, DA/eCB interactions may play a specialized role in ADHD patients to impact both motivation and attention. Impaired activity in both motivational and attentional networks typical in ADHD patients are stabilized by pharmacologically increasing brain DA concentration; furthermore, while cannabinoids generally negatively affect measures of impulsivity and attention, they uniquely enhance them in ADHD patients (Rubia et al., 2009; Cooper et al., 2017). Commonalities between motivation and attention in the preclinical setting may be found in the 5-CSRTT and impulsivity’s translatability to compulsive behavior, as cannabinergic regulation of this particular trait may be applied to constructs other than attentional dysfunction. For example, modulation of impulsivity through CB1 antagonism has been correlated with decreases in both alcohol and nicotine intake in rats, offering potential for therapies targeting eCB tone in addiction-related disorders (De Bruin et al., 2011). DA’s role in this broadly applicable trait is also noted. In humans, decreased D2/D3 binding and increased d-amphetamine-induced striatal DA release has been correlated with high levels of trait impulsivity and drug cravings (Buckholtz et al., 2010). Strongly modulated by both DA and eCBs, FSIs in the NAc are one candidate as a mediator of impulsive behavior through DA/eCB interaction, directly gating medium spiny neuron activity to regulate tracking of reward-predicting cues and inhibit premature responding (Caprioli et al., 2014; Wright et al., 2017; Pisansky et al., 2019; Boroujeni et al., 2020).

Generalizing this example of impulsivity to other behavioral components linked by motivation and attention, it is possible to conceive of various constructs impacted by both, and in turn, their susceptibility to eCB-modulated DA function. In this sense, interrogation of DA/eCB interactions within substrates known to modulate either motivation or attention might share explanatory potential across translatable constructs, particularly substance use disorders. Such studies may assess cannabinoid-induced changes to motivational and attentional processes through modifications of DA-mediated reward value signals, which have been shown to influence both types of measures. Yet, for clear dissociations between each measure, it will be important to characterize the separate neural representations contributing to their respective behavioral outputs. It is also important to note that while distinct measures of motivation and attention have been studied under conditions of cannabinoid exposure, there remains much to be learned about how these measures overlap within the context of DA/eCB interactions.

## Author Contributions

EO and DG conceptualized this review and equally shared in the majority of the writing. LH performed a secondary writing contribution that was lesser, but significant. All authors contributed to the article and approved the submitted version.

## Conflict of Interest

The authors declare that the research was conducted in the absence of any commercial or financial relationships that could be construed as a potential conflict of interest.
